# S1PR3-driven positive feedback loop sustains STAT3 activation and keratinocyte hyperproliferation in psoriasis

**DOI:** 10.1038/s41419-025-07358-w

**Published:** 2025-01-20

**Authors:** Panpan Lian, Li Li, Renwei Lu, Bin Zhang, Junaid Wazir, Chaode Gu, Bojie Ma, Wenyuan Pu, Wangsen Cao, Zhiqiang Huang, Zhonglan Su, Hongwei Wang

**Affiliations:** 1 State Key Laboratory of Analytical Chemistry for Life Science & Jiangsu Key Laboratory of Molecular Medicine, Medical School, Nanjing University, 210093 Nanjing, P.R. China; 2 Central Laboratory, Nanjing Chest Hospital, Affiliated Nanjing Brain Hospital, Nanjing Medical University, 210029 Nanjing, P.R. China; 3 Department of Dermatology, First Affiliated Hospital, Nanjing Medical University, 210029 Nanjing, P.R. China

**Keywords:** Lipid signalling, Receptor pharmacology

## Abstract

Psoriasis is a chronic inflammatory skin disorder characterized by hyperproliferation of keratinocytes and persistent inflammation. Although persistent activation of signal transducer and activator of transcription 3 (STAT3) is implicated in its pathogenesis, the mechanisms underlying the sustained STAT3 activation remain poorly understood. Here, we identify sphingosine-1-phosphate receptor 3 (S1PR3) as a critical regulator of STAT3 activation and psoriasis pathogenesis, orchestrating a self-amplifying circuit that sustains keratinocyte hyperproliferation and chronic inflammation. S1PR3 expression is markedly elevated in psoriatic lesions and correlates with disease severity. Using genetic and pharmacological approaches, we reveal a novel S1PR3–Src–STAT3 signaling axis that drives both early and prolonged STAT3 activation in keratinocytes. Mechanistically, S1PR3 operates through Gαi/PKA-mediated Src activation, enhancing STAT3 phosphorylation and subsequent transcriptional activity. Importantly, we reveal a previously unrecognized positive feedback loop wherein activated STAT3 directly upregulates S1PR3 expression, perpetuating inflammation and hyperproliferation. Genetic deletion of *S1pr3* in mice or pharmacological inhibition of S1PR3 significantly attenuates psoriasis-like skin inflammation, decreasing epidermal hyperplasia, dermal angiogenesis, and inflammatory mediator production. These findings provide new insights into the molecular mechanisms underlying psoriasis and identify S1PR3 as a promising therapeutic target. Our study suggests that disrupting the S1PR3–STAT3 feedback loop may offer a novel strategy for treating psoriasis and potentially other chronic inflammatory diseases driven by persistent STAT3 activation.

## Introduction

Psoriasis is a multifactorial, chronic, inflammatory skin disease with a global prevalence of 2–3% [1]. Clinical symptoms of psoriasis manifest as well-demarcated, and erythematous oval plaques with adherent silvery scales. Psoriasis is characterized by hyperproliferation, abnormal differentiation of keratinocytes, and infiltration of multiple inflammatory cells [2]. Keratinocytes participate in both the initiation and maintenance phases of psoriasis. As part of the innate immune system, keratinocytes can respond to multiple environmental triggers and release antimicrobial peptides to recruit immunocytes. During the chronic phase of psoriasis, keratinocytes produce a large number of chemokines, which amplify the inflammatory response.

STAT3 is a nuclear transcription factor that integrates signals from numerous receptors and nonreceptor tyrosine kinases. It plays a pivotal role in regulating cell proliferation, survival, apoptosis, and differentiation [3]. STAT3 is hyperactivated in the epidermis in psoriasis [4]. Transgenic mice with constitutively active STAT3 (K5. *Stat3*C) in keratinocytes can eventually develop psoriasiform lesions and pathological characteristics in skin tissue [5]. Moreover, the deletion of *Stat3* in keratinocytes but not in T cells reduced psoriasis-like dermatitis, indicating that persistent STAT3 activation in keratinocytes leads to the pathogenesis of psoriasis. Typically, STAT3 activation in response to IL-6 is rapid and transient. However, persistent STAT3 activation during skin inflammation is a distinguishing characteristic of psoriasis in patients and in imiquimod (IMQ)-induced psoriasiform dermatitis mouse model. The mechanisms leading to sustained STAT3 activation remain to be elucidated.

Sphingosine-1-phosphate (S1P), a bioactive sphingolipid metabolite, regulates lymphocyte trafficking, vascular integrity, and inflammation by binding to G protein-coupled S1P receptors (S1PRs) [6]. S1P has been implicated in various skin disorders, including psoriasis [7], atopic dermatitis [8], scleroderma [9], and other inflammatory diseases. It has been reported that the S1P level in the serum of psoriasis patients is increased [7], and S1PR1-3 is ubiquitously expressed [10]. Previous research has indicated that S1PR3 mediates NLRP3 inflammasome activation in macrophages [11] and that S1PR3 mediates the proinflammatory phenotype in myometrial cells [12]. However, the precise role of the S1P/S1PR pathway in psoriasis keratinocytes and its potential contribution to the sustained STAT3 activation observed in this disease remain largely unexplored.

In this study, we sought to elucidate the mechanisms underlying persistent STAT3 activation in psoriasis, with a focus on the potential role of sphingolipid signaling pathways. Our investigation focused on S1PR3, revealing its significant upregulation in psoriatic lesions and its correlation with disease severity. We identified a novel S1PR3-Src-STAT3 signaling axis and a positive feedback loop that maintains inflammatory and hyperproliferative states in psoriatic lesions. Through both genetic and pharmacological approaches, we explored the therapeutic potential of targeting S1PR3 in psoriasis.

## Materials and methods

### Clinical skin

Skin biopsy specimens were obtained from psoriasis patients and healthy subjects undergoing esthetic plastic surgery. All procedures were approved by the Clinical Research Ethics Committee of the First Affiliated Hospital of Nanjing Medical University, and informed consent was obtained from all participants.

### Animal studies

Six- to eight-week-old female BALB/c and C57BL/6 mice were treated with IMQ to develop the psoriasiform dermatitis, following previously published procedures [13]. In brief, the dorsal skin of the mice was shaved to expose a 2 cm × 3 cm area, and then 5% IMQ cream (Mingxinlidi, Sichuan) was applied to the dorsal skin for 6 consecutive days. All mice were randomly assigned to different groups. *S1pr3* knockout (KO) mice were purchased from GemPharmatech Company (strain no. T006548), in which exon 2 of the *S1pr3* gene was deleted via CRISPR/Cas9 technology. The genotyping primers amplified the KO fragment and wild-type (WT) fragment. The genotyping primers used were listed in Supplementary Table S1. All mice were kept in a specific pathogen-free environment. Psoriasis area and severity index (PASI) scores were obtained before the mice were euthanized. The spleen and skin were collected. The animal experiment was approved by Nanjing University Ethics Committee.

For keratinocyte-specific *S1pr3* knockdown mice, the mice were injected intracutaneously adeno-associated virus serotype 9 (AAV9) under the control of keratin 14 (*K14*) promoter. The shaved skin on the back was selected as the injection area. Five points were injected with K14-AAV9-shS1PR3 and K14-AAV9-shGFP with 50 μL per point. Each injection point was about 0.5 cm apart, and the injection depth was 1–2 mm. The needle was removed and the wounds were pressed with sterile cotton balls.

### Cell culture and treatments

Human immortalized epidermal keratinocyte (HaCaT) cells (KeyGen Biotech Company, SCSP-5091) were cultured in Dulbecco’s modified Eagle’s medium (Gibco) supplemented with 10% fetal bovine serum (Gibco) and 100 U/mL penicillin‒streptomycin (Thermo Fisher Scientific) at 37 °C in 5% CO_2_. Cells were treated with 25 μg/mL IL-6 (Sino Biological, 10395-HNAE) or 25 μg/mL IL-22 (Sino Biological, 13059-HNAE). Additional treatments included FTY720 (MedChemExpress, HY-12005), TY52156 (MedChemExpress, HY-19736), H89 dihydrochloride (MedChemExpress, HY-15979A), Rhosin hydrochloride (MedChemExpress, HY-12646), U73122 (MedChemExpress, HY-13419), Stattic (MedChemExpress, HY-13818), and Tirbanibulin (Selleck, S2700). Sodium orthovanadate (MedChemExpress, HY-D0852), Imiquimod (MedChemExpress, HY-B0180).

### Histopathology

Dorsal skin samples were fixed in 4% paraformaldehyde for 48 h and embedded in paraffin. After deparaffinization, the skin samples were cut into 10 μm-thick slices. These slides were then subjected to hematoxylin and eosin (H&E) staining for morphological analysis. Hyperkeratosis, parakeratosis, and Munro’s microabscess were evaluated in H&E-stained sections. Images were acquired at ×200 magnification using a Leica microscope system.

### Immunohistochemistry (IHC) and immunofluorescence (IF) staining

The skin sections were deparaffinized in dimethylbenzene and rehydrated in an ethanol solution. After antigen retrieval, the sections were incubated with the appropriate primary antibody at 4 °C overnight. For IHC, the sections were washed with PBS, followed by incubation with an HRP-conjugated secondary antibody for 1 h at room temperature. The sections were then developed with a DAB substrate and counterstained with hematoxylin.

For IF, the sections were washed with PBS and then incubated with a fluorescence-conjugated secondary antibody for 1 h at room temperature. The sections were mounted with a mounting medium containing DAPI and visualized using a fluorescence microscope. The primary antibodies used included anti-phospho-STAT3 (Tyr705) (Cell Signaling Technology, 9145), anti-STAT3 (Cell Signaling Technology, 12640), anti-Ki67 (Beyotime, AF1738), anti-Keratin 16 (Santa Cruz Biotechnology, sc-53255), anti-Keratin 17 (Cell Signaling Technology, 12509), and anti-CD130 (Thermo Fisher Scientific, PA5-28932).

### Masson’s trichrome (Masson’s) and periodic acid-Schiff (PAS) staining

For Masson’s trichrome staining, skin tissue samples were first fixed in Bouin’s solution, then deparaffinized in xylene and rehydrated through a graded series of alcohols to water. The sections were stained with Weigert’s iron hematoxylin for nuclei, followed by Biebrich scarlet-acid fuchsin for muscle fibers and cytoplasm. Phosphomolybdic-phosphotungstic acid differentiated these components and aniline blue stains collagen. Finally, the sections were dehydrated, cleared in xylene, and mounted. For PAS staining, skin tissues were fixed in formalin, deparaffinized in xylene, and rehydrated. Periodic acid oxidizes carbohydrates to aldehydes, which react with Schiff reagent to produce a magenta color. The sections were often counterstained with hematoxylin for nuclei, then dehydrated, cleared in xylene, and mounted.

### EdU proliferation assay

HaCaT cells were placed into six-well plates. When the confluence reached 70%, IL-6 and FTY720 (MedChemExpress, HY-12005) were coincubated with HaCaT cells for 24 h. Then, the HaCaT cells were stained with 20 μM 5-Ethynyl-2-deoxyuridine (EdU) for 2 h at 37 °C and visualized using a fluorescence microscope. EdU incorporation was detected using a commercial kit (Beyotime, C0071S) according to the manufacturer’s instructions.

### Cell transfection

The constitutively activated STAT3 plasmids were purchased from Addgene (STAT3-C Flag pRc/CMV, Addgene_8722). Plasmid DNA was purified using Endo-Free Plasmid Midi Kits (Omega Biotec, D6950). For siRNA transfection, S1PR3 siRNA or negative control siRNA was then transfected into HaCaT cells by using Lipofectamine™ RNAiMAX (Invitrogen, 13778030) according to the manufacturer’s guidelines. The siRNA sequences used are listed in Supplementary Table S1.

### Western blot analysis

Protein from HaCaT cells and skin tissue was extracted using RIPA lysis buffer (Beyotime, P0013B) supplemented with protease and phosphatase inhibitors. The protein concentration was measured with a BCA assay kit (Beyotime, P0009). Proteins (10–20 μg) were separated by 10% SDS‒PAGE and transferred to a PVDF membrane, which was subsequently blocked in 5% bovine serum albumin (BSA) for 1 h at room temperature. The PVDF membrane was incubated with the primary antibody at 4 °C overnight. The next day, the membranes were incubated with a secondary antibody conjugated with HRP (Bioworld, BS13278) for 1 h. Finally, enhanced chemiluminescence was used to visualize the protein levels using digital imaging systems. The following antibodies were used: anti-S1PR1 (Abcam, ab11424), anti-S1PR2 (Proteintech, 20442-1-AP), anti-S1PR3 (Abcam, ab108370), and anti-GAPDH (Bioworld, BS60630). Additionally, CD31 (Servicebio, GB11063-3-100), P-Src (Y419) (Abways, CY5468), Src (Abcam, ab32102), and anti-GAPDH (Fude, FD0063) antibodies were used.

### Quantitative RT‒polymerase chain reaction (qRT-PCR)

Total RNA was isolated from HaCaT cells and skin tissue using TRIzol reagent (Vazyme, R401-01). The isolated RNA was reverse transcribed into single-stranded cDNA with a reverse transcription kit (Vazyme, R302-01). All the quantitative PCR assays were performed with PowerUp SYBR Master Mix (Applied Biosystems, A25742). The relative quantity of mRNA was assessed by the 2^−^^ΔΔ^CT method. The sequences of the primers used are listed in Supplementary Table S1.

### Coimmunoprecipitation (Co-IP)

Cytosolic proteins were extracted from HaCaT cells by using a kit (Beyotime, P0027). Cytosolic lysates were incubated with a STAT3 antibody at 4 °C overnight to obtain bait proteins. The next day, the antibody-bound protein complex was captured using protein A or G beads. After washing with PBS for 3 times to remove nonspecific proteins, the precipitated proteins were eluted with 1× SDS loading buffer. The magnetic bead-antigen antibody complex was denatured at 95 °C for 5 min, and the supernatant was collected for detection by western blot.

### Online data analysis

The single-cell RNA sequencing (scRNA-seq) in psoriasis lesions and normal skin is sourced from an online repository (https://yz-studio.shinyapps.io/psoriaticskincellatlas2/) [14]. Additionally, the RNA-seq data containing 92 psoriatic and 82 normal skin samples was obtained from the Gene Expression Omnibus (GEO) database (GSE54456) [15]. The table with RPKM values was downloaded to examine the expression level of S1P-associated genes. For the Assay for Transposase-Accessible Chromatin (ATAC-seq), the chromatin accessibility data in psoriasis patients and healthy subjects were obtained (PRJNA597655) [16]. Using IGV software, the accessibility of the *K17* gene was observed in the skin of psoriasis patients and healthy subjects. Furthermore, publicly available chromatin immunoprecipitation sequencing (ChIP-seq) datasets (GSE25344, GSE175100, GSE230654, GSE250126, and GSE175125) were analyzed to identify an accessible chromatin configuration in the *S1PR3* promoter in keratinocytes and visualized using IGV software.

### RNA sequencing (RNA-seq)

RNA-seq was performed by the Beijing Genomics Institute using standard protocols. Briefly, RNA samples were denatured to open their secondary structure, and mRNA was enriched using oligo (dT)-attached magnetic beads. The RNA was then fragmented. First-strand cDNA was synthesized, followed by the synthesis of the second strand using dUTP. This was followed by end repair and the addition of a single ‘A’ nucleotide. The adaptors were ligated, and quality control was performed. This cDNA is then subjected to high-throughput sequencing technologies to generate millions of short-sequence reads. These reads were then aligned to a GRCh38/hg38 genome using bioinformatics tools HISAT2. Differential expression analysis was performed using DESeq2 to identify genes significantly altered between conditions.

### Liquid chromatography‒mass spectrometry (LC‒MS)

The gel pieces were subjected to SDS‒PAGE and destained for 20 min in 100 mM NH_4_HCO_3_ at 30%. The gel pieces were digested overnight in 12.5 ng/μl trypsin in 25 mM NH_4_HCO_3_. The peptides were extracted three times with 60% ACN/0.1% TFA. The extracts were pooled and dried completely by a vacuum centrifuge. The peptide of each sample was desalted on C18 cartridges (Empore™ SPE Cartridges, Sigma), concentrated by vacuum centrifugation, and reconstituted in 10 µl of 0.1% (v/v) formic acid. MS experiments were performed on a Q Exactive HF mass spectrometer that was coupled to an Easy nLC (Thermo Scientific). The MS data were analyzed using MaxQuant software version 1.5.8.3. MS data were searched against the UniProtKB Human database (157600 total entries, downloaded 07/2017). The database search results were filtered and exported with a < 1% false discovery rate (FDR) at the peptide and protein levels.

### Chromatin immunoprecipitation (ChIP) assays

HaCaT cells were cultured in 100-mm culture dishes and fixed with 1% formaldehyde for 15 min. After being fragmented, chromatin was mixed with protein A/G magnetic beads (MedChemExpress, HY-K0202). The STAT3 antibody and rabbit IgG were added to equal aliquots of chromatin and incubated at 4 °C overnight. The purified DNA from the input and immunoprecipitated samples was then subjected to PCR with *S1PR3* promoter-specific primers. The sequences of the *S1PR3* promoter-specific primers used are listed in Supplementary Table S1.

### Statistical analysis

The data are shown as the means ± standard deviations (SD). For data with a normal distribution, the statistical significance of the data was calculated by unpaired two-tailed Student-*t* test and one-way ANOVA in GraphPad Prism 8 software. Otherwise, a non-parametric Wilcoxon test or Kruskal–Wallis test was performed (*p < 0.05, **p < 0.01, or ***p < 0.001).

## Results

### Inhibition of S1PR alleviates IMQ-induced psoriasiform dermatitis in mice

**Fig. 1:**
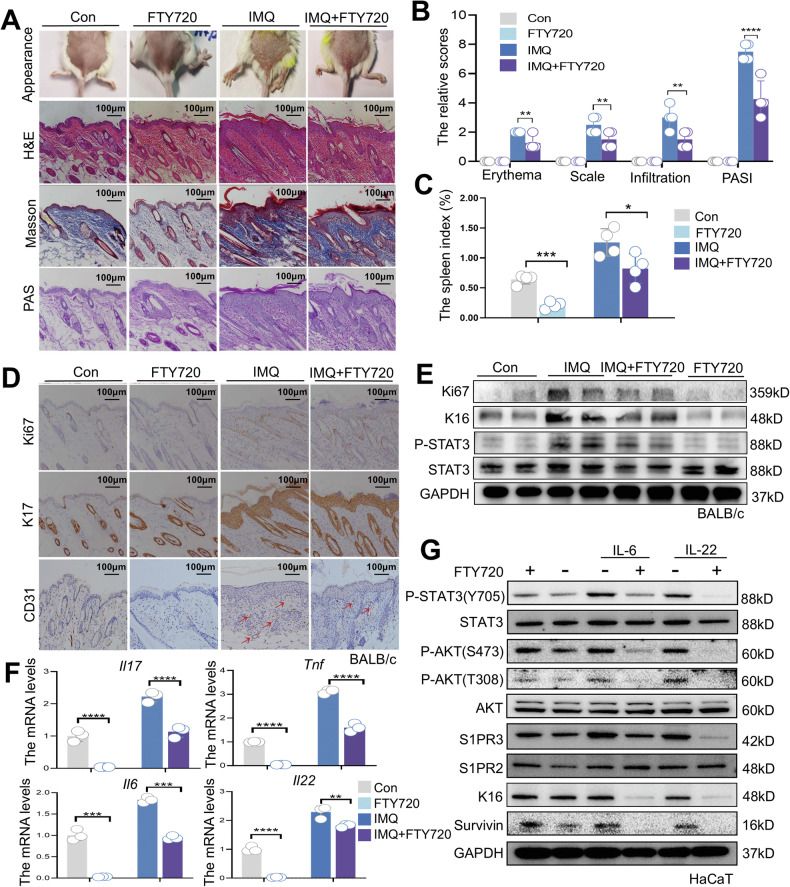
Inhibition of S1PR alleviates IMQ-induced psoriasiform dermatitis in mice. **A** Representative macroscopic images and histological staining (H&E, Masson’s trichrome, and PAS) of dorsal skin from mice treated with FTY720 (1 mg/kg/day, i.p.) or the solvent control vehicle prior to daily topical IMQ application. **B** PASI scores for assessing erythema, scaling, and skin thickness in IMQ-induced psoriatic lesions on day 7 (*n* = 4 mice per group). Higher scores indicate more severe psoriasis. **C** Spleen indices (spleen weight/body weight ratios) of control, IMQ-, and IMQ + FTY720-treated mice (*n* = 4 mice per group). **D** Representative immunohistochemistry images showing Ki67 and K17 expression in dorsal skin sections from control, FTY720-, IMQ-, and IMQ + FTY720-treated mice. **E** Western blot analysis of Ki67, K16, P-STAT3, STAT3, and GAPDH protein levels in dorsal skin lysates from control, IMQ-, IMQ + FTY720-, and FTY720-treated mice. **F** qRT‒PCR analysis of the mRNA levels of the proinflammatory cytokines *Il17*, *Tnf*, *Il6*, and *Il22* in the dorsal skin of control, IMQ, and IMQ + FTY720-treated mice (*n* = 3). **G** Western blot analysis of P-STAT3 (Y705), STAT3, P-AKT (S473), P-AKT (T308), AKT, S1PR3, S1PR2, K16, Survivin, and GAPDH protein levels in HaCaT cells stimulated with IL-6 or IL-22 in the presence or absence of FTY720 pretreatment. The data are presented as the mean ± SD. *p < 0.05, **p < 0.01, ***p < 0.001, ****p < 0.0001.

Studies have shown that serum levels of sphingosine-1-phosphate (S1P) are significantly elevated in patients with psoriasis [7]. To explore the consequences of S1P in psoriasis pathogenesis and the potential therapeutic relevance of modulating S1P receptors, we utilized FTY720, a pan-S1PR modulator approved for the treatment of multiple sclerosis [17]. Mice with IMQ-induced psoriasiform dermatitis were subjected to FTY720 (1 mg/kg/day) for 6 consecutive days. We found that FTY720 treatment alleviated clinical-associated pathological features, including acanthosis, parakeratosis, and epidermal thickening (Fig. 1A). It also suppressed keratinocyte hyperproliferation, as evidenced by reduced expression of Ki67, a marker of cellular proliferation (Fig. 1D, E). Additionally, staining of K16 and K17, markers of abnormal differentiation of keratinocytes, revealed reduced proliferation and differentiation following FTY720 treatment (Fig. 1D, E). FTY720 treatment suppressed inflammatory responses, as indicated by a decreased spleen index (Fig. 1C) and reduced expression of the proinflammatory cytokines interleukin-17 (*Il17*), tumor necrosis factor (*Tnf*), interleukin-6 (*Il6*), and interleukin-22 (*Il22*) in lesional skin (Fig. 1F). Notably, we found FTY720 treatment significantly decreased STAT3 activation and its downstream Survivin in vitro or in vivo. (Fig. 1E, G). Given that S1PR1-3 is ubiquitously expressed in many organs, we investigated the expression of S1PR1-3 in IL-6-induced keratinocytes after FTY720 treatment. Intriguingly, FTY720 selectively downregulated S1PR3 without affecting S1PR2 expression (Fig. 1G), suggesting that its therapeutic effects were primarily mediated through S1PR3. Taken together, these findings indicate that S1PR inhibition alleviates IMQ-induced psoriasiform dermatitis, coinciding with reduced STAT3 activation.

### S1PR3 is selectively upregulated in psoriatic keratinocytes and correlates with disease severity

**Fig. 2:**
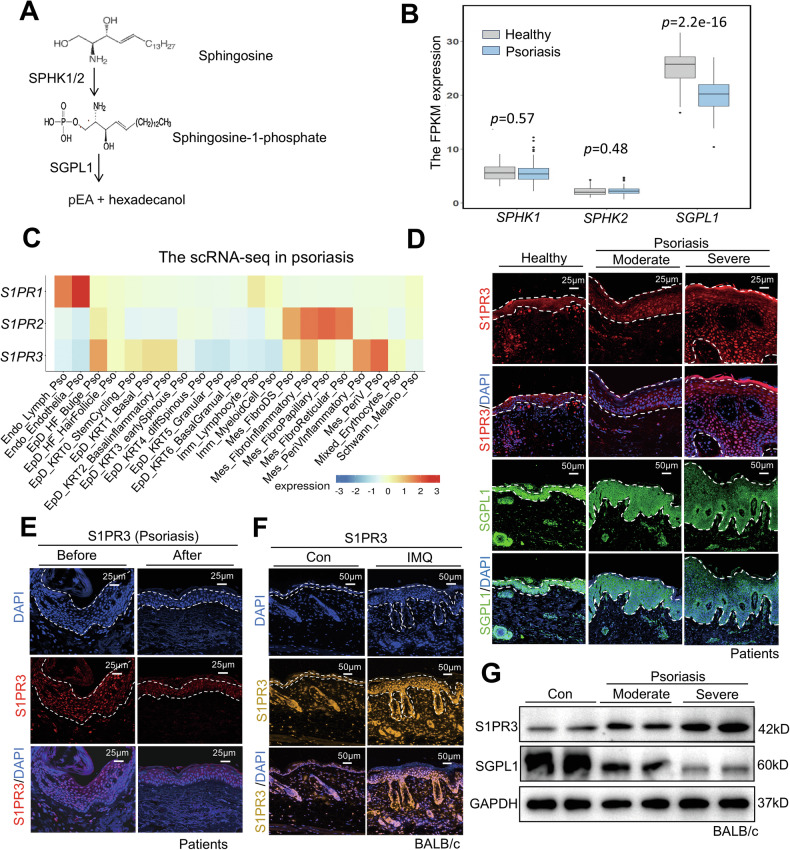
S1PR3 expression is elevated in psoriatic lesions and correlates with disease severity. **A** Schematic illustration of S1P generation and degradation mediated by SPHK1/2 and SGPL1. **B** RPKM values of *SPHK1*, *SPHK2*, and *SGPL1* in normal and psoriatic skin from the GSE54456 dataset. *p* values are indicated for each comparison. **C** Heatmap showing *S1PR1*, *S1PR2*, and *S1PR3* expression across different cell types in psoriatic lesions based on publicly available single-cell RNA sequencing data. **D** Representative immunofluorescence images of S1PR3 (red) and SGPL1 (green) in healthy, moderate, and severe psoriatic skin. Nuclei were counterstained with DAPI (blue). Scale bars: 20 μm. **E** Immunofluorescence staining of S1PR3 (red) in patients before and after psoriasis treatment. Nuclei were counterstained with DAPI (blue). Scale bars: 25 μm. **F** Immunofluorescence staining of S1PR3 (yellow) in the skin of control mice and mice with IMQ-induced psoriasiform dermatitis. Nuclei were counterstained with DAPI (blue). Scale bars: 20 μm. **G** Western blot analysis (top) and quantification (bottom) of S1PR3 and SGPL1 expression in control and IMQ-treated mouse skin. The data are presented as the mean ± SD. ****p < 0.0001.

S1P levels are regulated by sphingosine kinase 1 (SPHK1)- and SPHK2-mediated production and sphingosine-1-phosphate lyase 1 (SGPL1)-mediated degradation (Fig. 2A). To investigate S1P levels in psoriasis, we analyzed the expression of key S1P regulatory genes (*SPHK1*, *SPHK2*, and *SGPL1*) using the psoriasis dataset GSE54456. Our investigation revealed intriguing patterns: while the expression of *SPHK1* and *SPHK2* remained unchanged, we observed a marked downregulation of *SGPL1* in psoriatic lesions compared to healthy controls (Fig. 2B). This observation is consistent with previous reports of elevated S1P levels in psoriasis patients [7], suggesting a shift toward increased S1P signaling in this disease.

Further examination of S1P signaling in psoriasis using publicly available single-cell RNA-sequencing data revealed distinct localization patterns of S1P receptors in psoriatic lesions (https://yz-studio.shinyapps.io/psoriaticskincellatlas2/). Analysis revealed that *S1PR3* was mainly localized to the epidermal layer, whereas *S1PR2* and *S1PR1* were primarily expressed in dermal fibroblasts and endothelial cells, respectively (Fig. 2C). Immunofluorescence analysis of clinical samples confirmed the S1PR3 in psoriatic epidermis, was positively correlated with the severity of psoriasis and inversely correlated with SGPL1 expression in psoriatic lesions (Fig. 2D), as supported by increased S1PR3 expression and decreased SGPL1 expression in server psoriasis. Conversely, S1PR3 expression was downregulated after anti-psoriasis treatment (Fig. 2E), indicating the important role of S1PR3 expression in the progression of psoriasis. To validate these findings in vivo, we employed an IMQ-induced psoriasiform dermatitis mouse model. Consistent with the clinical data, IMQ-treated mice exhibited markedly increased S1PR3 expression in the epidermal layer, as evidenced by both immunofluorescence and western blot analyses (Fig. 2F). Furthermore, we investigated the association between S1PR3 and SGPL1 in IMQ-induced mice. SGPL1 expression decreased with the severity of psoriasis, whereas S1PR3 expression increased, showing that S1PR3 was negatively related to SGPL1 expression and was positively related to psoriasis severity in IMQ-induced mice (Fig. 2G). Interestingly, S1PR1 expression was decreased in human psoriatic lesions but elevated in the IMQ model (Fig. S1A–E). Taken together, these findings highlight the differential expression patterns of S1P receptors in psoriasis and underscore the specific association of the expression of S1PR3, which is predominantly expressed in keratinocytes, with the severity of psoriasis.

### S1PR3 knockout mitigates IMQ-induced psoriasiform dermatitis in mice

**Fig. 3:**
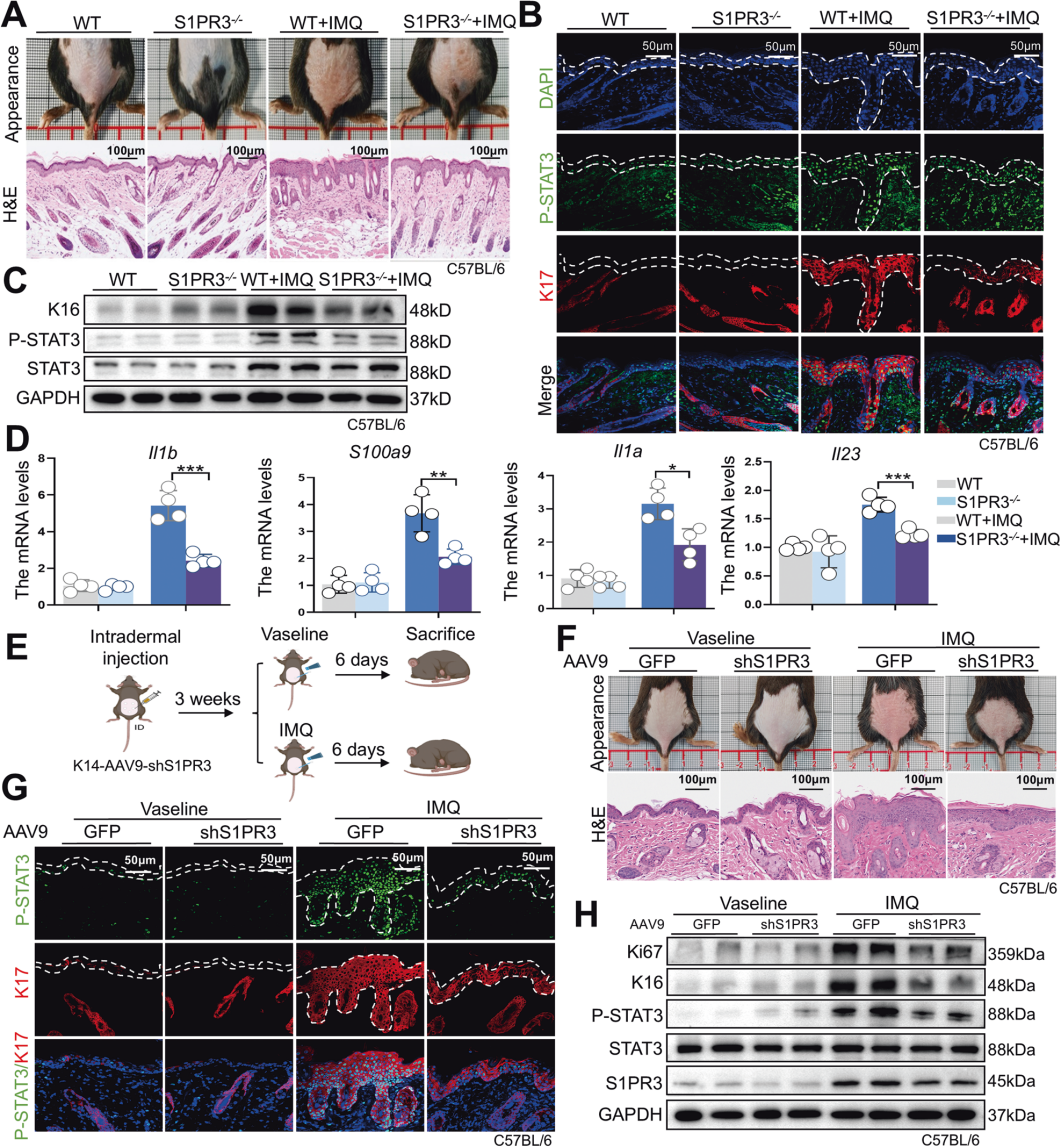
S1PR3 knockout attenuates IMQ-induced psoriasiform dermatitis. **A** Macroscopic appearance of dorsal skin (top row), skin-draining lymph nodes (middle row), and histological staining (H&E) of the back skin of WT mice and S1PR3-KO mice with and without IMQ treatment. Scale bars: 100 μm. **B** Representative immunofluorescence images of K17 (red) and P-STAT3 (green) in psoriasis-like lesions from WT and S1PR3*-*KO mice with and without IMQ treatment. Nuclei were counterstained with DAPI (blue). Scale bars: 50 μm. **C** Western blot analysis of K16, P-STAT3, and total STAT3 expression in lesional skin from WT and S1PR3*-*KO mice with and without IMQ treatment. **D** qRT‒PCR analysis of key psoriasis-associated inflammatory mediators (*Il1b*, *S100a9*, *Il1a*, and *Il23*) in the skin of WT, S1PR3-KO, IMQ-treated WT and S1PR3*-*KO mice (*n* = 4). **E** Schematic illustration of keratinocyte-specific S1PR3 knockdown workflow by AAV9-shS1PR3 intradermal injection. **F** Macroscopic appearance of dorsal skin, and histological staining (H&E staining) of the back skin of WT mice and KC-S1PR3-KD mice with and without IMQ treatment. **G** Representative immunofluorescence images of K17 (red) and P-STAT3 (green) in psoriatic lesions from WT and S1PR3*-*KO mice with and without IMQ treatment. **H** Western blot analysis of Ki67, K16, P-STAT3, STAT3, and S1PR3 expression in the lesion skin from WT and S1PR3*-*KO mice with and without IMQ treatment. The data are presented as the mean ± SD. *p < 0.05; **p < 0.01; ***p < 0.001.

To elucidate the role of S1PR3 in psoriasis pathogenesis, we subjected S1PR3*-*KO mice to IMQ for 7 days. S1PR3*-*KO mice displayed a marked attenuation of the phenotypic features of psoriasis, as evidenced by macroscopic examination (Fig. 3A). Histological analysis revealed that the epidermal thickness was inhibited after S1PR3 knockout, as evidenced by H&E staining (Fig. 3A). Furthermore, S1PR3*-*KO markedly improved psoriasis-related phenotypes, including erythema, scaling, and infiltration, resulting in a reduced PASI scores (Fig. S2A). IMQ-induced psoriasiform dermatitis is often accompanied by splenomegaly and dermal angiogenesis, which are thought to contribute to the infiltration of inflammatory cells into the skin [18]. Notably, compared with their WT counterparts, S1PR3*-*KO mice exhibited a significant reduction in spleen size (Fig. S2B), suggesting that S1PR3 plays a crucial role in regulating the systemic inflammatory response. These findings collectively demonstrate that S1PR3 is a key orchestrator of psoriasiform dermatitis.

### S1PR3 knockout inhibits hyperproliferation and inflammatory response in mice

Given the known involvement of STAT3 activation and keratinocyte hyperproliferation in psoriasis, we examined the expression of phosphorylated STAT3 (P-STAT3) and K17, a marker of keratinocyte activation, in the epidermis. Immunofluorescence and western blot analyses revealed decreased levels of P-STAT3 and K17, which are markers of keratinocyte activation, in the epidermis of S1PR3*-*KO mice (Fig. 3B, C). Importantly, staining for Ki67 revealed a marked reduction in the number of proliferating keratinocytes in the epidermis of the S1PR3*-*KO mice compared to that in the epidermis of the WT controls following IMQ treatment (Fig. S2C). Immunofluorescence staining for CD31, an endothelial cell marker, revealed a decrease in dermal angiogenesis and capillary formation in the S1PR3*-*KO mice (Fig. S2C). Consistent with the observed phenotypic improvements, S1PR3-KO mice exhibited a significantly attenuated inflammatory response. qRT‒PCR analysis revealed reduced expression of critical pro-inflammatory mediators, including *Il1b*, *Il1a*, *Il23*, and *S100a9*, in the lesional skin of IMQ-treated S1PR3*-*KO mice (Fig. 3D). These findings collectively demonstrate that S1PR3 regulates multiple pathogenic processes, including keratinocyte hyperproliferation, STAT3 activation, dermal angiogenesis, and the production of inflammatory mediators.

To further investigate the role of keratinocyte-specific S1PR3 in psoriasis progression, we generated keratinocyte-specific S1PR3knockdown mice through intradermal injection of adeno-associated virus serotype 9-shS1PR3 with the *K14* promoter (K14-AAV9-shS1PR3) (Fig. 3E). Our results indicated that keratinocyte-specific S1PR3 knockdown (KC-S1PR3-KD) alleviated the psoriatic lesions in IMQ-induced mice, as evidenced by a reduction in macroscopic appearance following K14-AAV9-shS1PR3 injection (Fig. 3F). H&E staining revealed a reduction in epidermal thickness in KC-S1PR3-KD mice treated with IMQ, compared to that in control mice **(**Fig. 3F). The psoriasis-related clinical features (erythema, scaling, infiltration, and PASI) were significantly decreased in the lesions of IMQ-treated KC-S1PR3-KD mice (Fig. S2D). Keratinocyte S1PR3 knockdown did not influence the inflammatory responses, as reflected by the similar spleen index in Fig. S2E. Furthermore, histological analysis revealed a significant reduction in the expression of Ki67, K17, and K16, which are markers associated with keratinocyte hyperproliferation and aberrant differentiation, in the epidermis of IMQ-treated KC-S1PR3-KD mice (Fig. 3G, H). Notably, the activation of STAT3 was significantly reduced by keratinocyte S1PR3 knockdown, compared with that in IMQ-induced mice (Fig. 3G), which was further confirmed by western blot analysis (Fig. 3H). In summary, these findings demonstrate that keratinocyte S1PR3 is closely related to the development of psoriasis.

### STAT3 activation abolishes the S1PR3 inhibition-mediated anti-proliferation

**Fig. 4:**
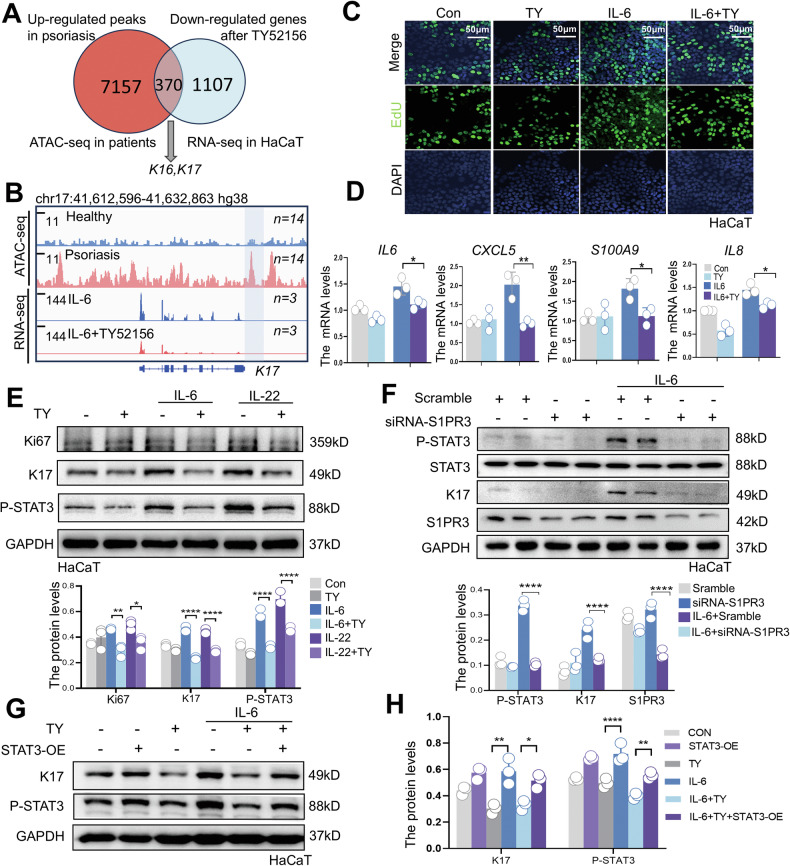
STAT3 activation abolishes the S1PR3 inhibition-mediated anti-proliferation. **A** Venn diagram showing overlap between upregulated ATAC-seq peaks in psoriasis patients and downregulated genes after TY treatment in HaCaT cells (10 μM TY and 25 μg/mL IL-6), including K16 and K17. **B** IGV visualization of K17 expression in psoriasis patients via ATAC-seq (*n* = 14) (PRJNA597655) and in HaCaT cells treated with IL-6 or IL-6 plus TY via RNA-seq (*n* = 3). **C** EdU staining demonstrates that TY inhibits IL-6-induced HaCaT cells proliferation. Scale bars: 20 μm. **D**. qRT‒PCR analysis of inflammatory mediators and chemokines (*IL6*, *CXCL5*, *S100A9*, and *IL8*) in HaCaT cells cotreated with IL-6 and TY for 24 h. **E** Western blot analysis and quantification of Ki67, K17, and P-STAT3 expression in HaCaT cells treated with TY and IL-6 or IL-22. **F** Western blot analysis and quantification of P-STAT3, STAT3, K17, and S1PR3 in HaCaT cells transfected with S1PR3 siRNA and treated with IL-6 for 24 h. **G**, **H** Western blot analysis (**G**) and quantification (**H**) of K17 and P-STAT3 in HaCaT cells transfected with the STAT3C plasmid and then treated with IL-6 alone or in combination with TY. The data are presented as the mean ± SD. *p < 0.05; **p < 0.01; ***p < 0.001; ****p < 0.0001.

We conducted a comparative analysis between ATAC-seq data from psoriasis patients and the transcriptomic changes induced by TY52156 (hereafter referred to as TY) treatment in vitro (Fig. 4A). Our analysis revealed 370 psoriasis-upregulated genes that were suppressed by TY, including the key hyperproliferation markers K16 and K17 (Fig. 4A, B), suggesting a direct anti-hyperproliferative effect on keratinocytes after S1PR3 inhibition. S1PR3 inhibition significantly reduced keratinocyte proliferation and suppressed the expression of critical inflammatory mediators, including *IL6*, *IL8*, *CXCL5*, and *S100A9* (Fig. 4C, D). These findings confirm that S1PR3 signaling modulates both keratinocyte hyperproliferation and the inflammatory microenvironment characteristic of psoriatic lesions.

To elucidate whether the improved psoriasiform dermatitis observed in *S1pr3-*KO mice was due to a reduction in STAT3 in keratinocytes, we then investigated the mechanistic link between S1PR3 and STAT3 signaling in HaCaT keratinocytes. TY treatment significantly reduced the IL-6- and IL-22-induced phosphorylation of STAT3 at Y705, a key residue for STAT3 activation (Fig. 4E). Moreover, prolonged S1PR3 inhibition suppressed keratinocyte hyperproliferation and abnormal differentiation (Fig. 4E). Additionally, regarding the mouse model of psoriasis established by IMQ application, we treated HaCaT cells with the IMQ compound (hereafter referred to as R837). Our results revealed that R837 treatment also upregulated P-STAT3 levels indirectly. Consistent with the outcome induced by IL-6, S1PR3 antagonist TY significantly suppressed IMQ-induced hyperproliferation and STAT3 activation (Fig. S3A). The knockdown of S1PR3 corroborated these findings and significantly impaired IL-6-induced STAT3 activation and K17 expression (Fig. 4F). To establish a causal relationship between STAT3 activation and the antiproliferative effects of TY, we expressed a constitutively active STAT3 mutant (STAT3C) in keratinocytes. This genetic manipulation abrogated the anti-hyperplastic effects of TY (Fig. 4G), demonstrating that STAT3 inhibition was critical for the therapeutic effects of S1PR3 antagonism. These data reveal a pivotal role for the S1PR3-STAT3 axis in driving keratinocyte hyperproliferation and sustaining the inflammatory environment in psoriasis.

### Src mediates S1PR3-induced biphasic STAT3 activation in keratinocytes via Gαi/PKA signaling

**Fig. 5:**
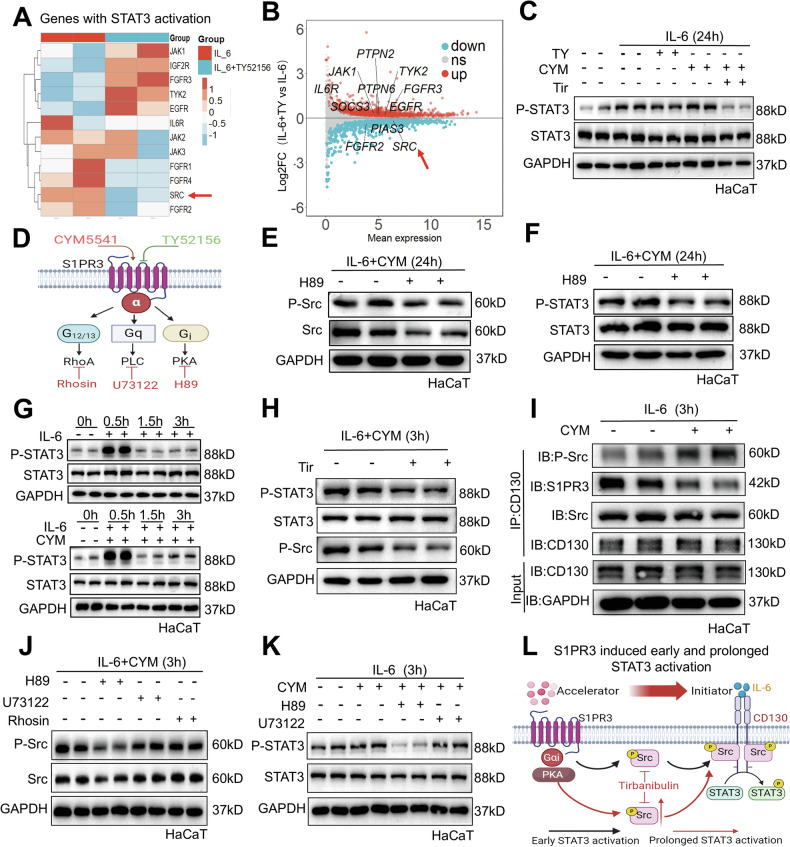
Src mediates S1PR3-induced biphasic STAT3 activation in keratinocytes via Gαi/PKA signaling. **A** Heatmap of positively regulated STAT3 genes from RNA-seq of HaCaT cells treated with IL-6 or IL-6 + TY for 24 h. **B** MA plot of STAT3 regulatory genes. Src is highlighted with a red arrow. **C** Western blot showing that Src inhibition by Tri (100 nM) blocked CYM-induced P-STAT3 activation at 24 h. **D** Schematic of S1PR3 coupled with Gα proteins and their respective inhibitors. **E**, **F** Western blot of P-Src and Src (**E**) and P-STAT3 and STAT3 (**F**) in HaCaT cells treated with IL-6 + CYM and 5 μM H89 for 24 h. **G** Time course of STAT3 activation in HaCaT cells treated with IL-6 alone (top) or IL-6 plus 10 μM CYM (bottom) for 0, 0.5, 1.5, or 3 h. **H** Src inhibition by Tri reduces STAT3 activation after treatment with IL-6 plus CYM for 3 h. **I** Co-IP assays showing interactions between P-Src, S1PR3, and CD130 in HaCaT cells treated with IL-6 or IL-6 plus CYM for 3 h. **J**, **K** Western blot of P-Src and Src (**J**), and P-STAT3 and STAT3 (**K**) after treatment with H89, U73122, or Rhosin, showing the differential effects of various inhibitors on Src and STAT3 activation. **L** Schematic representation of the role of S1PR3 as an accelerator of IL-6-induced early and prolonged STAT3 activation via Gαi/PKA/Src signaling. The data are presented as the mean ± SD. *p < 0.05, **p < 0.01, ***p < 0.001.

**Fig. 6:**
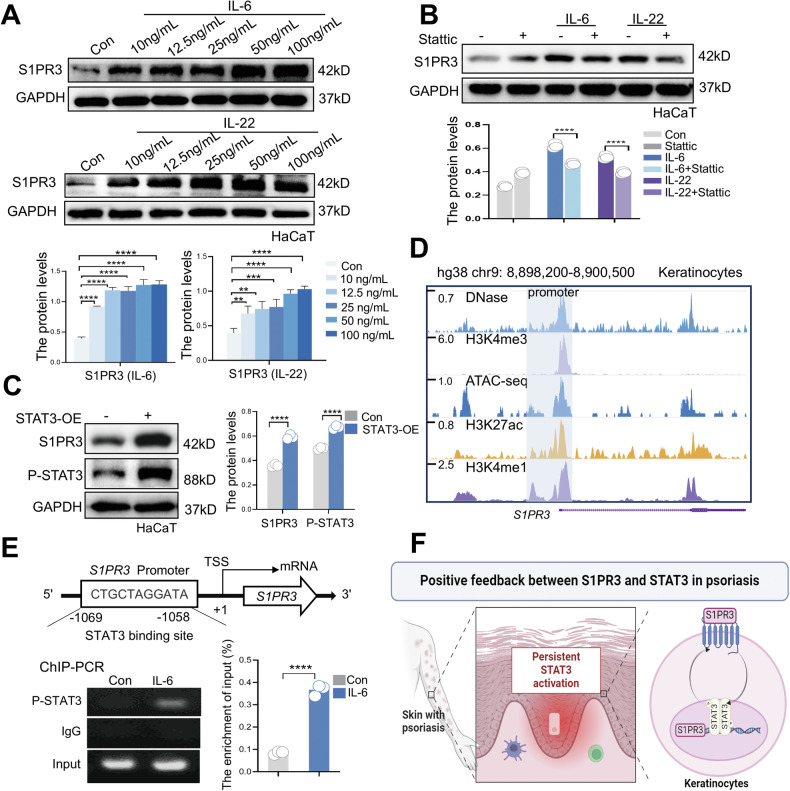
STAT3 activation drives a positive feedback loop by directly upregulating S1PR3 expression. **A** Western blot analysis of S1PR3 expression in HaCaT cells treated with increasing concentrations of IL-6 (top) or IL-22 (bottom). The quantification of S1PR3 protein levels is shown below each blot. **B** Western blot and quantification of S1PR3 expression in HaCaT cells treated with the STAT3 inhibitor Stattic (10 μM) in the presence or absence of 25 μg/mL IL-6 or 25 μg/mL IL-22. **C** Immunoblotting and quantification of S1PR3 and P-STAT3 levels in HaCaT cells transfected with a constitutively active STAT3 (STAT3C) plasmid for 48 h. **D** Chromatin accessibility profile of the S1PR3 promoter region in keratinocytes showing DNase hypersensitivity and histone modifications from ChIP-seq data in the GEO database. **E** Schematic representation of the predicted (JASPER) STAT3 binding site in the S1PR3 promoter (top). ChIP‒PCR analysis confirming STAT3 binding to the S1PR3 promoter in response to IL-6 treatment (bottom). **F** Schematic diagram illustrating the positive feedback loop between S1PR3 and STAT3 in psoriatic keratinocytes. The data are presented as the mean ± SD. *p < 0.05; **p < 0.01; ***p < 0.001; ****p < 0.0001.

To elucidate the mechanism underlying the observed reduction in STAT3 activation upon *S1pr3* knockdown, we performed RNA sequencing of HaCaT keratinocytes treated with IL-6 alone or in combination with TY. Gene expression analysis following S1PR3 inhibition revealed the upregulation of STAT3 negative regulators (*PTPN2*, *PTPN6*, and *SOCS3*) and downregulation of STAT3 positive regulators (*Src*, *FGFR2*) (Figs. 5A, S3B). Notably, MA plot analysis revealed *Src* as a highly expressed gene among the downregulated positive STAT3 regulators (Fig. 5B). Moreover, the inhibition of Src with Tirbanibulin (hereafter referred to as Tri) blocked the prolonged STAT3 activation induced by the S1PR3 agonist CYM5541 (hereafter referred to as CYM) (Fig. 5C). Additionally, to rule out the phosphatase-mediated STAT3 activation, we utilized sodium orthovanadate (hereafter referred to as SOV), a pan-phosphatase inhibitor, to treat HaCaT cells. Our results indicated that sodium orthovanadate treatment did not influence the P-STAT3 levels in HaCaT cells, compared to IL-6 and CYM treatment (Fig. S3C), suggesting that phosphatase was not involved in the S1PR3-mediated STAT3 activation in psoriasis. To identify the specific G protein subtype involved in S1PR3-mediated STAT3 activation, we used inhibitors for Gαi/PKA (H89), Gαq/PLC (U73122), and Gα12/13/RhoA (Rhosin hydrochloride) (Fig. 6D). Selective inhibition of Gαi/PKA, but not Gαq/PLC or Gα12/13/RhoA, suppressed both Src and STAT3 activation under IL-6 plus CYM stimulation (Figs. 5D–F, S3D). This delineated the specific G-protein subtype (Gαi) and downstream effector (PKA) involved in S1PR3-Src-STAT3 signaling.

Intriguingly, we found that S1PR3 activation not only prolonged but also accelerated STAT3 activation in IL-6-treated keratinocytes (Fig. 5G). This early activation was also Src-dependent, as Tri blocked this effect (Fig. 5H). Notably, CYM alone did not activate STAT3 (Fig. S3E), indicating that S1PR3 acts as an amplifier of IL-6-mediated STAT3 activation rather than an independent activator. These results suggest that S1PR3 functions as a molecular rheostat, fine-tuning both the early and sustained phases of STAT3 signaling. Mechanistically, we found increased binding of phosphorylated Src to gp130 in IL-6 plus CYM-treated keratinocytes (Fig. 6I), providing a molecular basis for the enhanced STAT3 activation. The critical role of the Gαi/PKA/Src pathway was further reinforced by the observation that H89 (a Gαi/PKA inhibitor) blocked both early Src (Y419) and STAT3 (Y705) activation (Fig. 5J, K), while U73122 and Rhosin had no effect (Fig. S3F). Notably, this S1PR3-induced STAT3 activation was not associated with changes in STAT3 ubiquitination or stability (Fig. S3G–K), suggesting that the enhanced STAT3 activation is primarily due to increased phosphorylation rather than altered protein turnover. In summary, we revealed a novel signaling axis in which S1PR3, through Gαi/PKA, activates Src to enhance both early and sustained STAT3 activation in keratinocytes (Fig. 5L).

### STAT3 activation drives a positive feedback loop by directly upregulating S1PR3 expression

The observed persistence of STAT3 activation and elevated S1PR3 levels in psoriatic keratinocytes led us to hypothesize a potential positive feedback mechanism. Treatment of HaCaT keratinocytes with increasing concentrations of IL-6 or IL-22, known activators of STAT3, led to a dose-dependent increase in S1PR3 expression (Fig. 6A). Importantly, this induction was blocked by the STAT3 inhibitor Stattic (Fig. 6B). Furthermore, overexpression of constitutively active STAT3 (STAT3C) in HaCaT cells resulted in marked upregulation of S1PR3 (Fig. 6C), further supporting a close association between STAT3 activation and S1PR3 expression. To elucidate the molecular basis of this regulation, we analyzed publicly available ChIP-seq datasets, which revealed an accessible chromatin configuration at the *S1PR3* promoter in keratinocytes (Fig. 6D). In silico analysis using the JASPAR database (https://jaspar.elixir.no/) identified a putative STAT3 binding site within the *S1PR3* promoter (Fig. 6E). ChIP‒PCR assays confirmed significant STAT3 enrichment at this site in IL‒6-stimulated keratinocytes (Fig. 6E), providing direct evidence for STAT3-mediated transcriptional regulation of *S1PR3*. These findings reveal a positive feedback loop in which STAT3 activation directly upregulates S1PR3 expression, which in turn enhances STAT3 activation. This self-reinforcing circuit likely contributes to the persistent inflammation and keratinocyte hyperproliferation observed in psoriatic lesions (Fig. 6F).

### S1PR3 inhibition alleviates psoriasis-like lesions in IMQ-treated mice

**Fig. 7:**
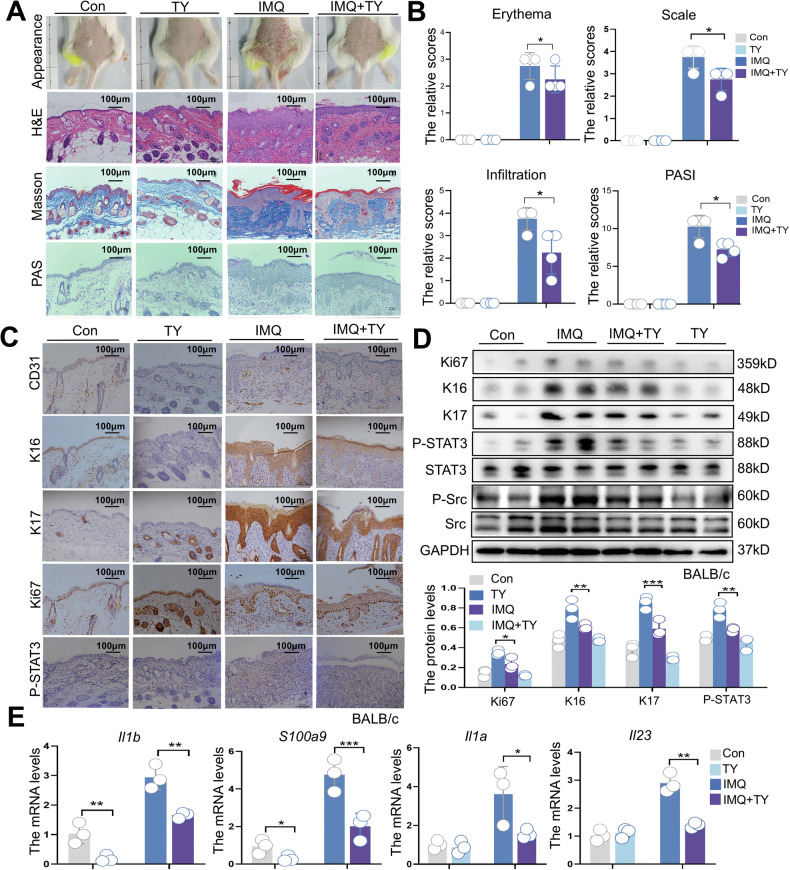
S1PR3 inhibition alleviates psoriasis-like lesions in IMQ-treated mice. **A** Representative macroscopic images and histological staining (H&E, Masson’s trichrome, and PAS) of mouse dorsal skin treated with TY (3 mg/kg/day) or the solvent control prior to daily topical application of IMQ (62.5 mg/day). **B** PASI scores for assessing erythema, scaling, and skin thickness in IMQ-induced psoriasis-like lesions (*n* = 4). Higher scores indicate more severe psoriasis. **C** Representative immunohistochemistry images showing the expression of CD31, K16, K17, Ki67, P-STAT3, and Src in dorsal skin sections from mice treated with solvent control, IMQ, IMQ + TY, or TY alone. **D** Western blot analysis of Ki67, K16, K17, P-STAT3, STAT3, P-Src, and Src protein levels in dorsal skin lysates from mice treated with solvent control, IMQ, IMQ + TY, or TY alone. GAPDH served as a loading control. **E** qRT‒PCR analysis of the mRNA levels of the psoriasis-associated proinflammatory mediators *Il1b*, *S100a9*, *Il1a*, and *Il23* in mouse skin (*n* = 3). The data are presented as the mean ± SD. *p < 0.05, **p < 0.01, ***p < 0.001, ****p < 0.0001.

**Fig. 8:**
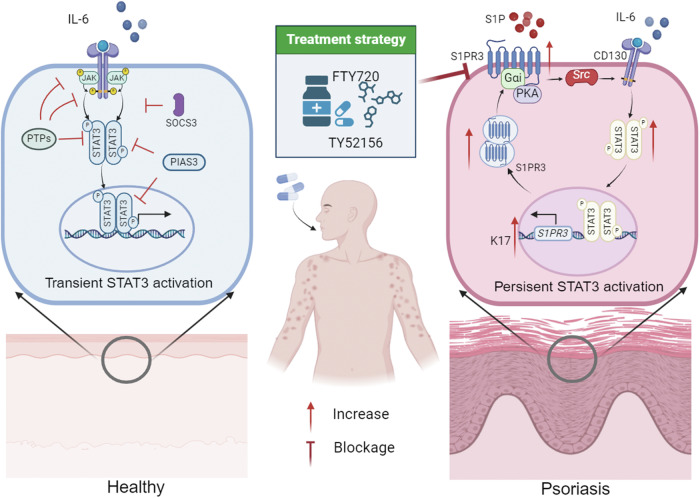
Proposed model of S1PR3-mediated keratinocyte hyperproliferation in psoriasis. A schematic illustration depicts the role of S1PR3 and STAT3 in psoriasis pathogenesis. We found that S1PR3 expression was markedly elevated in psoriatic lesions. Mechanistically, we identified a novel S1PR3-Src-STAT3 signaling axis that drives both early and sustained STAT3 activation in keratinocytes through Gαi/PKA signaling. Additionally, activated STAT3 binds to the S1PR3 promoter and upregulates its expression, creating a positive feedback loop to perpetuate the hyperproliferation and inflammatory state in psoriasis.

To evaluate the therapeutic potential of S1PR3 inhibition in psoriasis, we administered the S1PR3 antagonist TY (3 mg/kg/day) to mice with IMQ-induced psoriasiform dermatitis for six consecutive days. This treatment significantly ameliorated the severity of lesions and reduced epidermal thickness in the dorsal skin, as evidenced by both visual assessment and histological analysis (Fig. 7A). TY treatment markedly improved psoriasis-related clinical features, including erythema, scaling, and infiltration, resulting in a reduced PASI score (Fig. 7B). Histological examination further revealed no significant collagen deposition or glycogen accumulation in the lesions following TY treatment (Fig. 7A). Furthermore, we observed a significant reduction in the expression of markers associated with keratinocyte hyperproliferation and aberrant differentiation (Ki67, K17, and K16) in the epidermis following TY treatment (Fig. 7C, D). Moreover, S1PR3 inhibition reduced dermal angiogenesis, as evidenced by decreased CD31 staining (Fig. 7C). TY treatment downregulated key inflammatory mediators, including *Il1b*, *Il1a*, *S100a9*, and *Il23* (Fig. 7E), with a little side effect on the immune response of the control mice. Crucially, S1PR3 inhibition reduced both Src and STAT3 activation in vivo (Fig. 7D), corroborating our in vitro findings and supporting the S1PR3–Src–STAT3 signaling axis as a key driver of psoriasis pathogenesis. These findings collectively demonstrate that targeting the S1PR3–Src–STAT3 axis through selective S1PR3 antagonism effectively alleviates psoriasis-like inflammation (Fig. 8).

## Discussion

Psoriasis is characterized by hyperproliferation and aberrant differentiation of keratinocytes in the epidermis, accompanied by the expression of K16 and K17 cytoskeletal proteins [19]. Uncontrolled STAT3 activation is strongly implicated in the development of psoriasis. The activation of STAT3 is commonly regulated by the IL-6 or IL-22 subfamily produced by Th17 cells, ultimately contributing to epidermal hyperplasia and inflammation [20, 21]. However, the pathological mechanism underlying the induction of aberrant STAT3 activation is unknown.

Since the levels of sphingosine-1-phosphate derived from ceramide and sphingolipid metabolism are increased in psoriasis, we identified increased S1PR3 as a key receptor closely related to the severity of psoriatic lesions in this study. S1PR3 KO in vitro and in vivo inhibited keratinocyte proliferation and STAT3 phosphorylation, confirming the role of S1PR3 in STAT3 activation and the pathogenesis of psoriasis. Notably, S1PR3 participated in early STAT3 activation, as indicated by advanced STAT3 activation by the S1PR3 agonist at 3 h. This result prompted us to hypothesize that other metabolites and receptors further accelerate and sustain STAT3 activation. Here, we revealed that S1PR3 was involved in both early and prolonged STAT3 activation in keratinocytes. The increased expression of S1PR3 was the major cause of persistent STAT3 activation in psoriasis keratinocytes, suggesting that S1PR3 and STAT3 are involved in interactions between sphingolipid metabolites and abnormal proliferation in psoriasis patients. The discrepancy of S1PR1 expression in IMQ-induced mice and psoriasis patients was observed in our study. The discrepancy of S1PR1 expression in IMQ-induced mice and psoriasis patients was observed in our study. Psoriasis in humans often manifests as a chronic condition in patients, whereas the IMQ-induced mice model primarily reflects an acute inflammatory state. Notably, S1PR1 has been shown to be upregulated in various acute inflammatory conditions, including stem cells of myeloid leukemia and LPS-induced acute lung injury [22, 23], suggesting the pivotal role of S1PR1 in maintaining acute inflammation. These findings indicate that S1PR1 expression may vary depending on the stage of the inflammatory response, with higher expression levels potentially associated with acute phases of inflammation, as observed in the IMQ model.

Regarding the association between S1PR3 and STAT3, we found Src was the major intermediate of S1PR3-mediated STAT3 activation. Src inhibition by Tri blocked S1PR3-induced early and prolonged STAT3 activation. Activated S1PR3 increased the levels of P-Src, which then bound to gp130 of IL-6R during S1PR3-mediated early STAT3 activation without affecting Src expression. Regarding prolonged STAT3 activation, we found that activated S1PR3 upregulates the expression of Src and P-Src, thereby maintaining sustained STAT3 activation in the lesion. To further explore the link between S1PR3 and Src activation, we discovered that the Gαi subunit couples with S1PR3 to initiate STAT3 signaling [24]. Gαi primarily modulates cAMP levels and PKA activity, while Gαq-activated phospholipase C (PLC) β and Gα_12/13_ can activate small GTPase families [25–27]. In our study, inhibition of Gαi/PKA by H89 blocked S1PR3-associated Src and STAT3 activation but did not activate Gαq/PLC or Gα_12/13_/Rho. It was reported that cAMP/PKA and increased phosphorylation of Src at serine were found 40 years ago [28], after which a consensus PKA site at serine 17 of Src (S17) was found [29]. A recent study showed that Src activation at S17 by PKA could subsequently induce Src (Y419) phosphorylation [30]. In addition, the literature indicates that GPCRs contribute to Src activation independently of the classical G protein, wherein β-arrestins (βarrs) combined with activated GPCRs act as adaptor proteins to assemble Src. The GPCR-β-arrestin complex can allosterically activate the proto-oncogene kinase Src [31, 32]. It has been reported that the recruitment of GPCRs is crucial for the regulation of intracellular signaling cascades [33], including MAPK/ERK [34] and AKT [35, 36]. Here, we found that S1PR3, a GPCR subfamily member, activated the STAT3 signaling pathway.

FTY720, a pan-S1PR modulator also known as fingolimod, was the first FDA-approved drug used to treat multiple sclerosis [17]. Here, we demonstrated that FTY720 treatment ameliorated the manifestations of psoriasis (erythema, scaling, and infiltration) and reduced the synthesis of inflammatory cytokines (*Il22*, *Il23*, and *Tnf*), showing anti-inflammatory effects and anti-proliferative effects. These results aligned with the immunosuppressive role of FTY720 in decreasing the number of circulating lymphocytes from lymphoid organs and the Th17-mediated inflammatory response [37–39]. However, we found that FTY720 inhibited the inflammatory response in control mice, as shown in Fig. 1C and F. FTY720 was reported to bind to S1PR1-5 with a similar affinity as S1P, except for S1PR2 [40]. Our findings demonstrated that the protective role of FTY720 in psoriasis was mediated by internalized S1PR3, highlighting the significance of S1PR3 in the pathogenesis of psoriasis. Furthermore, we found that S1PR3 inhibition by TY significantly reduced abnormal differentiation and hyperproliferation in keratinocytes, with minimal side effects on the immune system of control mice.

## Conclusion

In this study, we found that S1PR3 expression was closely related to the severity of psoriasis. S1PR3 knockout alleviated the manifestations and pathological features of psoriasis by inhibiting the hyperproliferation and inflammatory response. Mechanistically, S1PR3 was involved in the early or prolonged activation of STAT3 in psoriatic lesions by regulating the Gαi/PKA/Src pathway to sustain keratinocyte hyperproliferation and chronic inflammation. In addition, the persistent activation of STAT3 in psoriasis was attributed to elevated S1PR3 in the epidermal skin, resulting in positive feedback between S1PR3 and STAT3. Targeting the S1P/S1PR3/STAT3 axis might serve as a potential therapeutic strategy for patients with psoriasis.

## Supplementary information

Supplemental Material

## Data Availability

The data that support the findings of this study are available upon request from the corresponding author.
