# Supplementary material for: S1PR3-driven positive feedback loop sustains STAT3 activation and keratinocyte hyperproliferation in psoriasis
Source: Cell Death Dis. 2025 Jan 20;16(1):31. doi: 10.1038/s41419-025-07358-w (PMC11746942; doi:10.1038/s41419-025-07358-w)

SUPPLEMENTARY INFORMATION

Lian et al. 2024

Fig S1

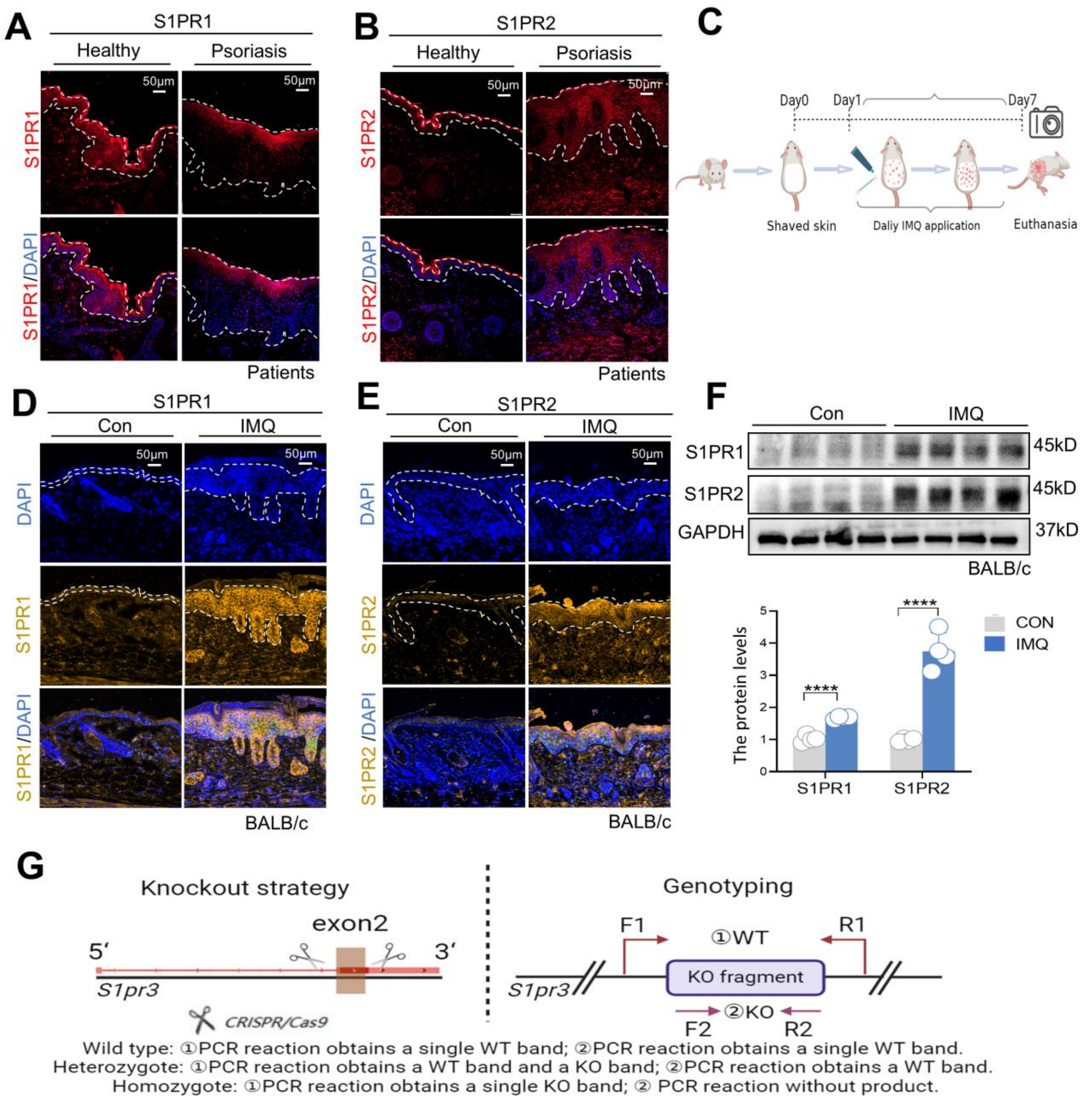

Fig S1. S1PR1 and S1PR2 expression in psoriatic skin and imiquimod (IMQ)-induced mouse skin. A, B. Representative immunofluorescence images showing S1PR1 (**A**) and S1PR2 (**B**) expression (red) in healthy and psoriatic human skin. Nuclei were counterstained with DAPI (blue). Scale bars: 50 μm. **C.** Schematic of the IMQ-induced psoriasis-like mouse model. Mice were treated daily with topical IMQ (62.5 mg) on the shaved dorsal skin for 6 consecutive days to induce psoriasis-like skin inflammation. Skin samples were collected on day 7 for analysis. **D, E**

Representative immunofluorescence images of S1PR1 (**D)** and S1PR2 (**E)** expression (red) in dorsal skin sections from control and IMQ-treated mice. Nuclei were counterstained with DAPI (blue). Scale bars: 50 μm (upper panels) and 20 μm (lower panels). **F.** Western blot analysis (left) and quantification (right) of S1PR1 and S1PR2 protein levels in dorsal skin lysates from control and IMQ-treated mice (n=4). **G.** Schematic of the KO strategy used to generate *S1pr3*-KO mice. Exon 2 of the *S1pr3* gene was deleted by CRISPR Cas9. Genotyping was performed by PCR using primers specific for the WT, and KO alleles. The data are presented as the mean ± S.D. *$p < 0.05$, **$p < 0.01$, ***$p < 0.001$, ****$p < 0.0001$.

Fig S2

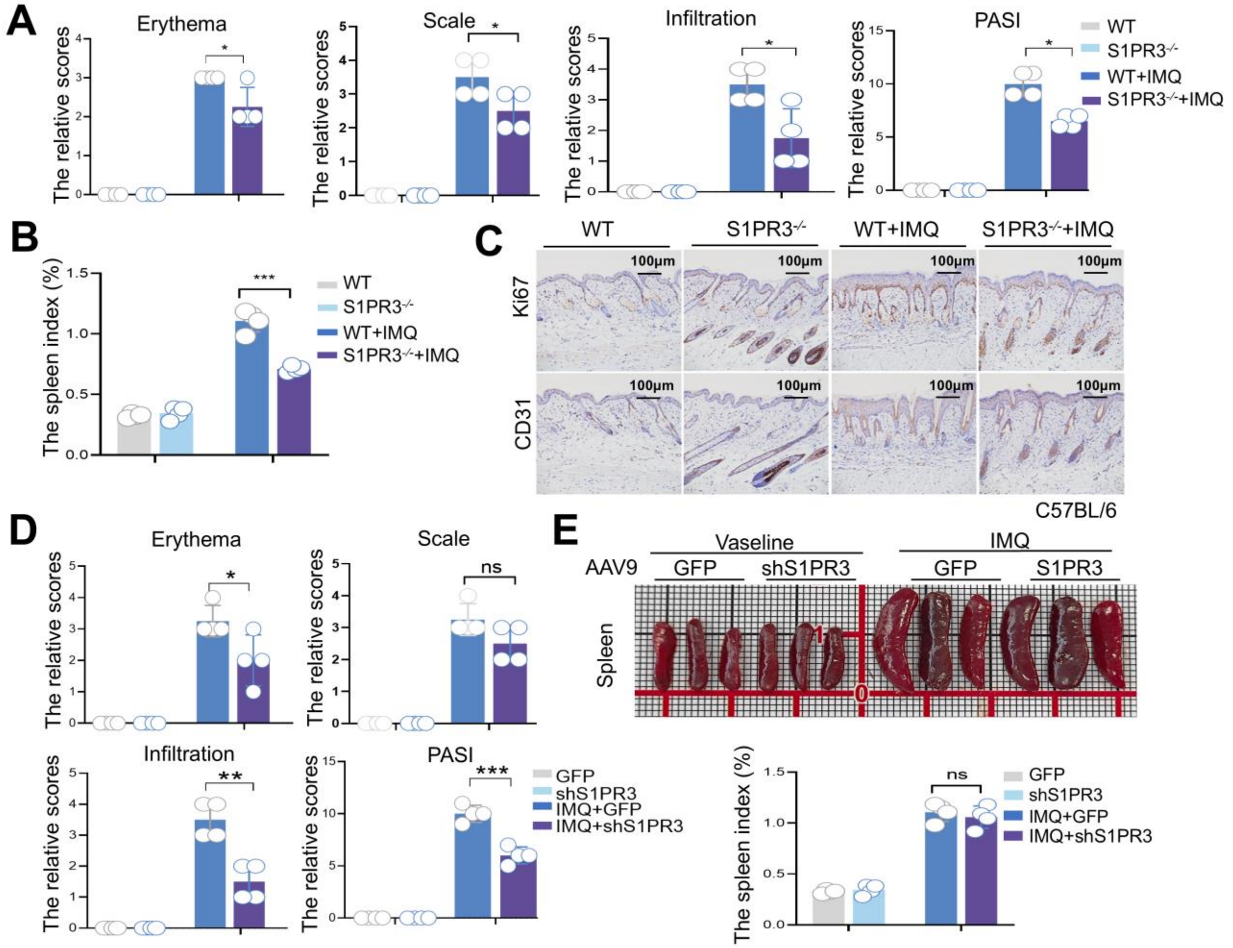

Fig S2. Keratinocytes-specific S1PR3 knockdown alleviates the psoriatic lesions in IMQ-induced mic.

A. PASI scores for assessing erythema, scaling, and skin thickness in IMQ-induced psoriatic lesions on day 7 (*n*=4). **B.** Quantification of the spleen indices of WT and S1PR3-KO mice treated with or without IMQ (*n*=4). **C.** IHC staining of Ki67 (top row) and CD31 (bottom row) in the back skin of WT and S1PR3-KO mice with and without IMQ treatment. Scale bars: 100 μm. **D**. PASI scores for assessing erythema, scaling, and skin thickness in IMQ-induced psoriatic lesions on day 7 (*n*=4). **E.** Quantification of the spleen indices of WT and S1PR3-KO mice treated with or without IMQ (*n*=4). The data are presented as the mean ± S.D. *$p < 0.05$, **$p < 0.01$, ***$p < 0.001$, ****$p < 0.0001$.

Fig S3

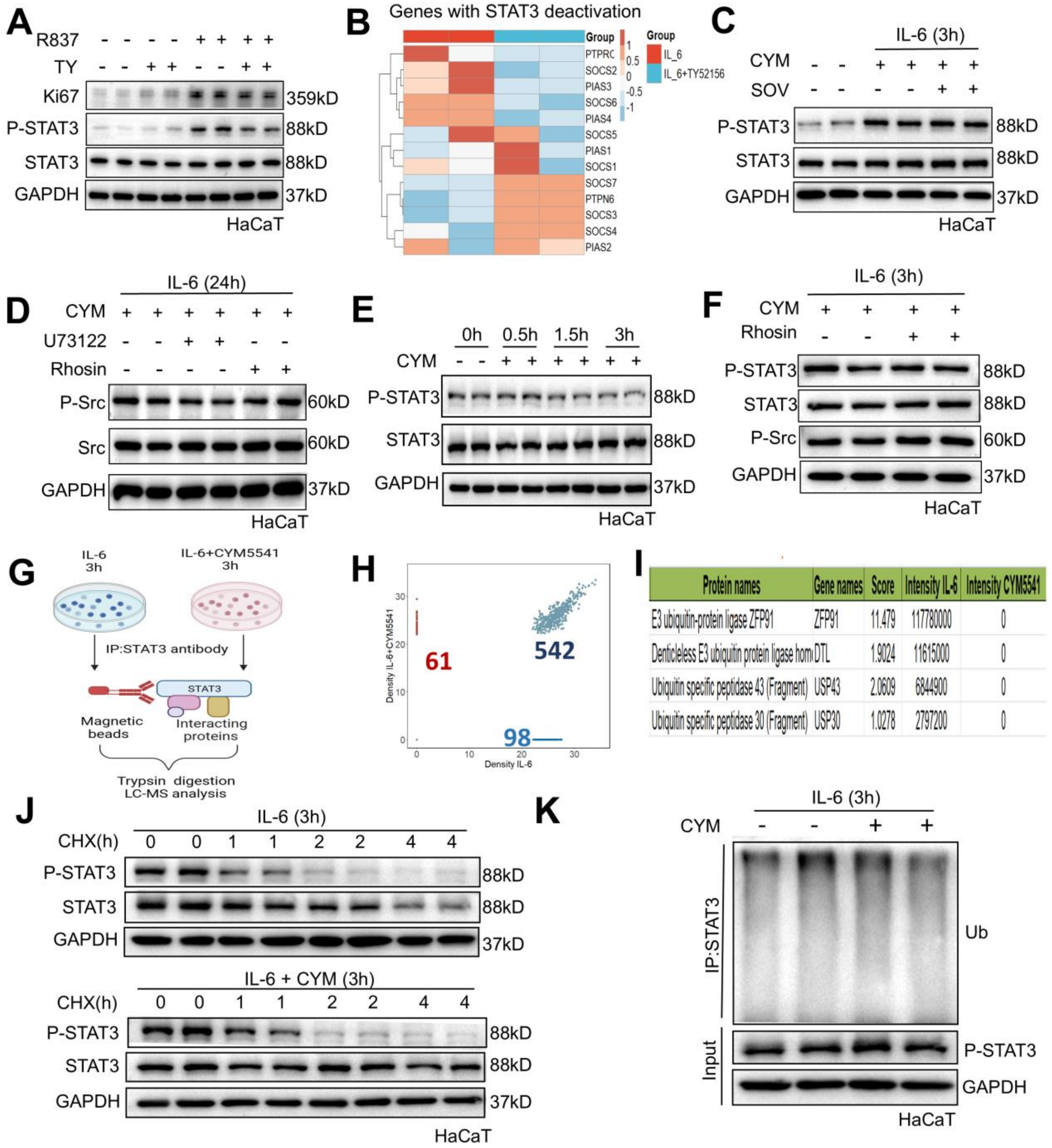

Fig S3. STAT3 ubiquitination is not associated with S1PR3-mediated STAT3 activation in keratinocytes.

A. Western blot analysis of P-STAT3, STAT3, Ki67 expression in HaCaT cells treated with 20 μM imiquimod R837 for 24 hours or 2 hours. **B.** Heatmap showing the expression of genes negatively regulating STAT3 activation from RNA-seq data of HaCaT cells treated with IL-6 (24 h) or IL-

6+TY52156 (24 h). The color scale represents the Z scores. **C.** Western blot analysis of P-STAT3 and STAT3 expression in HaCaT cells treated with 1mM SOV (sodium orthovanadate, a pan-phosphatase inhibitor) for 3 hours. **D**. Western blot analysis of Src expression in HaCaT cells treated with 1 μM U73122 (a PLC inhibitor) and 2 μM Rhosin (a Rho inhibitor) for 24 hours. GAPDH served as a loading control.**E.** Western blot analysis of P-STAT3, STAT3, and GAPDH in HaCaT cells pretreated with 10 μM CYM (an S1PR3 agonist) for the indicated time points (0 h, 0.5 h, 1.5 h, and 2 h). **F.** Western blot analysis of P-STAT3, STAT3, P-Src, and GAPDH in HaCaT cells treated with IL-6 (3 h) in the presence or absence of 2 μM Rhosin and 10 μM CYM. **G.** Schematic of the immunoprecipitation-mass spectrometry (IP-MS) workflow used to identify STAT3-interacting proteins in IL-6-stimulated (3 h) HaCaT cells treated with or without CYM. **H.** Venn diagram showing the number of P-STAT3-interacting proteins identified by IP-MS. A total of 701 proteins were identified, 98 of which were dissociated from P-STAT3 upon CYM treatment. **I.** Table listing the E3 ubiquitin ligases found among the 98 proteins that dissociated from P-STAT3 after CYM treatment, along with their gene names, scores, and intensity values with or without CYM. **J.** Western blot analysis of P-STAT3, STAT3, and GAPDH in HaCaT cells treated with 10 μM cycloheximide (CHX) for the indicated time points (0, 1, 2, 4 h) to assess protein stability. Cells were pretreated with IL-6 (3 h) or IL-6+CYM (3 h) prior to CHX addition. **K.** Coimmunoprecipitation (Co-IP) of STAT3 followed by Western blot analysis of ubiquitin (Ub) and STAT3 in HaCaT cells treated with IL-6 or CYM for 3 hours. Input shows Western blot analysis of P-STAT3 and GAPDH in whole-cell lysates. The data are presented as the mean ± S.D. *$p < 0.05$, **$p < 0.01$, ***$p < 0.001$, ****$p < 0.0001$.

Supplementary table 1: Primers used in this study

RT-qPCR primers	Forward Primer (5'-3')	Reverse Primer (5'-3')
**Mouse**		
Il6	TAGTCCTTCCTACCCCAATTTCC	TTGGTCCTTAGCCACTCCTTC
Il22	ATGAGTTTTTCCCTTATGGGGAC	GCTGGAAGTTGGACACCTCAA
Il17a	TGACCCCTAAGAAACCCCCA	TCATTGTGGAGGGCAGACAA
Il1b	GCAACTGTTCCTGAACTCAACT	ATCTTTTGGGGTCCGTCAACT
Tnf	CAGGCGGTGCCTATGTCTC	CGATCACCCCGAAGTTCAGTAG
Ifng	ACAGCAAGGCGAAAAAGGATG	TGGTGGACCACTCGGATGA
Actb	AGCAAGCAGGAGTACGATGA	GGTGTAAAACGCAGCTCAGTAA
S100a9	ACTCTTTAGCCTTGAAGAGCAAG	TTCTTGCTCAGGGTGTCAGG
Il23	CAAAGGATCCGCCAAGGTCT	GGAGGTGTGAAGTTGCTCCA
Il1a	CGAAGACTACAGTTCTGCCATT	GACGTTTCAGAGGTTCTCAGAG
**Human**		
IL6	CCACCGGGAACGAAAGAGAA	GAGAAGGCAACTGGACCGAA
CXCL5	AGCGCGTTGCGTTTGTTTAC	TGGCGAACACTTGCAGATTAC
CXCL8	AAGGTGCAGTTTTGCCAAGG	CCCAGTTTTCCTTGGGGTCC
S100A9	GGTCATAGAACACATCATGGAGG	GGCCTGGCTTATGGTGGTG
KRT17	GCCGCATCCTCAACGAGAT	CGCGGTTCAGTTCCTCTGTC
EGF	TGTCCACGCAATGTGTCTGAA	CATTATCGGGTGAGGAACAACC
**Primers for *S1pr3*-KO mice**	**Forward Primer (5'-3')**	**Reverse Primer (5'-3')**
PCR①_WT	AGGACAGTTCGAGTTCCTTCCTC	TCTCTTGTTTGCGTGACAGAGC
PCR②_KO	TCTTATGTCCGGCAGGAAGACG	CAATCACTACGGTCCGCAGAAG
**S1PR3 siRNA**	**Forward Primer (5'-3')**	**Reverse Primer (5'-3')**
S1PR3	CAUUCUGAUGUCCGGUAGGUU	UUGUAAGACUACAGGCCAUCC
Scramble	UUCUCCGAACGUGUCACGU	ACGUGACACGUUCGGAGAA
**ChIP-qPCR**	**Forward Primer (5'-3')**	**Reverse Primer (5'-3')**
S1PR3 promoter	TCAGCCAGTATCCTAGCAGTAA	ACAACCAGGATAACAATACAGC

Original raw western blots

Figure 1E

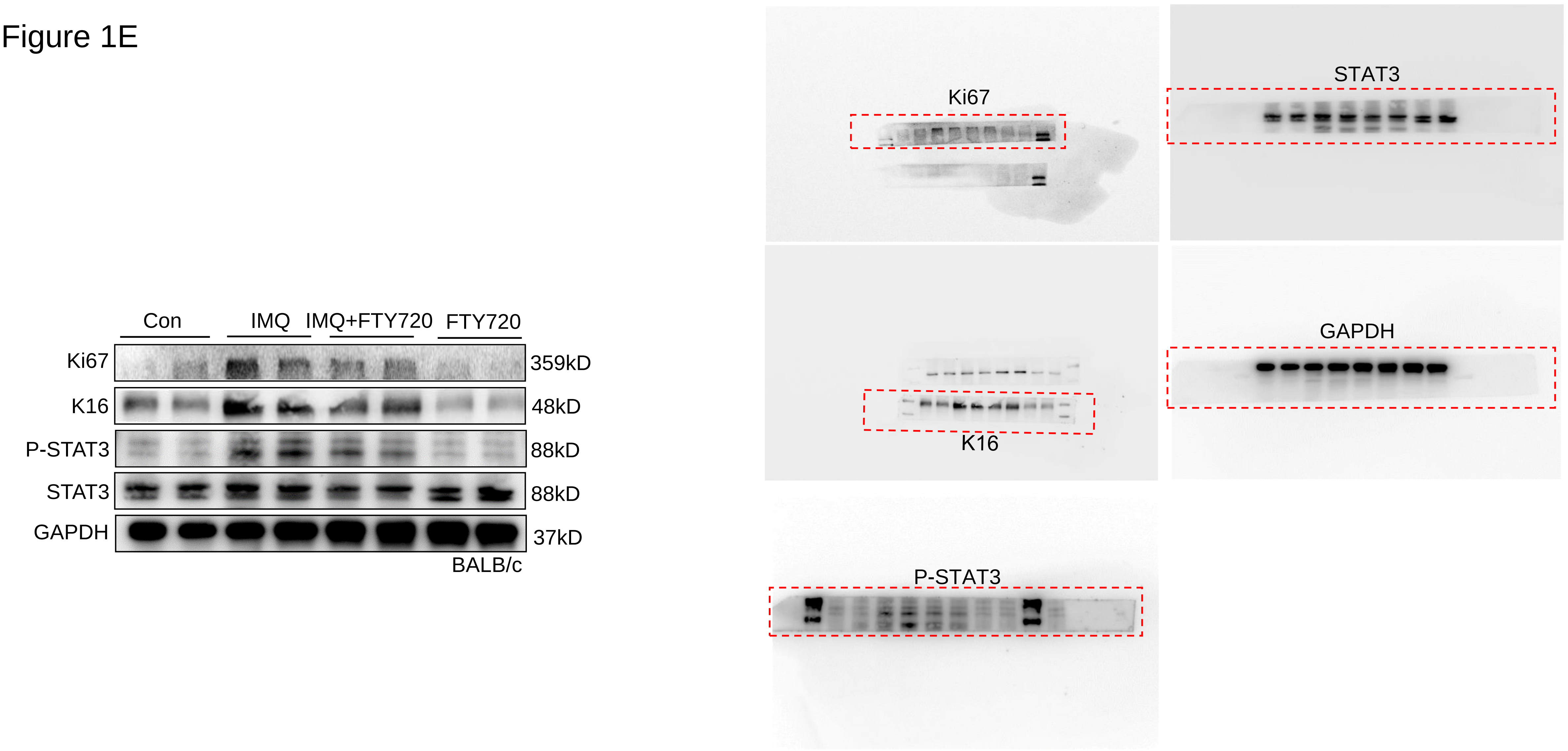

Figure 1G

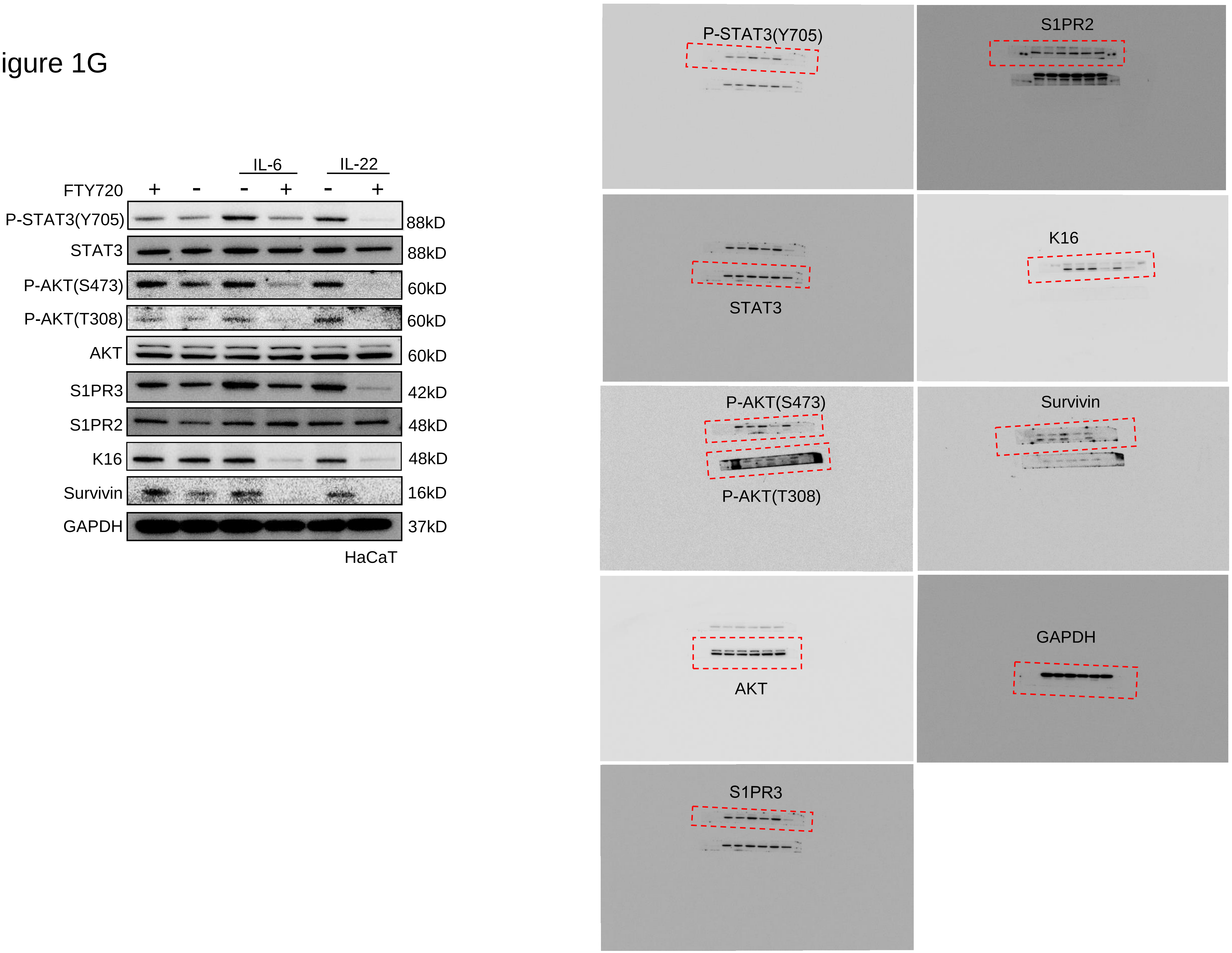
IL-6
IL-22
FTY720 + - - + - +
P-STAT3(Y705)
STAT3
P-AKT(S473)
P-AKT(T308)
AKT
S1PR3
S1PR2
K16
Survivin
GAPDH
88kD
88kD
60kD
60kD
60kD
42kD
48kD
48kD
16kD
37kD
HaCaT
P-STAT3(Y705)
S1PR2
STAT3
K16
P-AKT(S473)
P-AKT(T308)
Survivin
AKT
GAPDH
S1PR3

Figure 2G

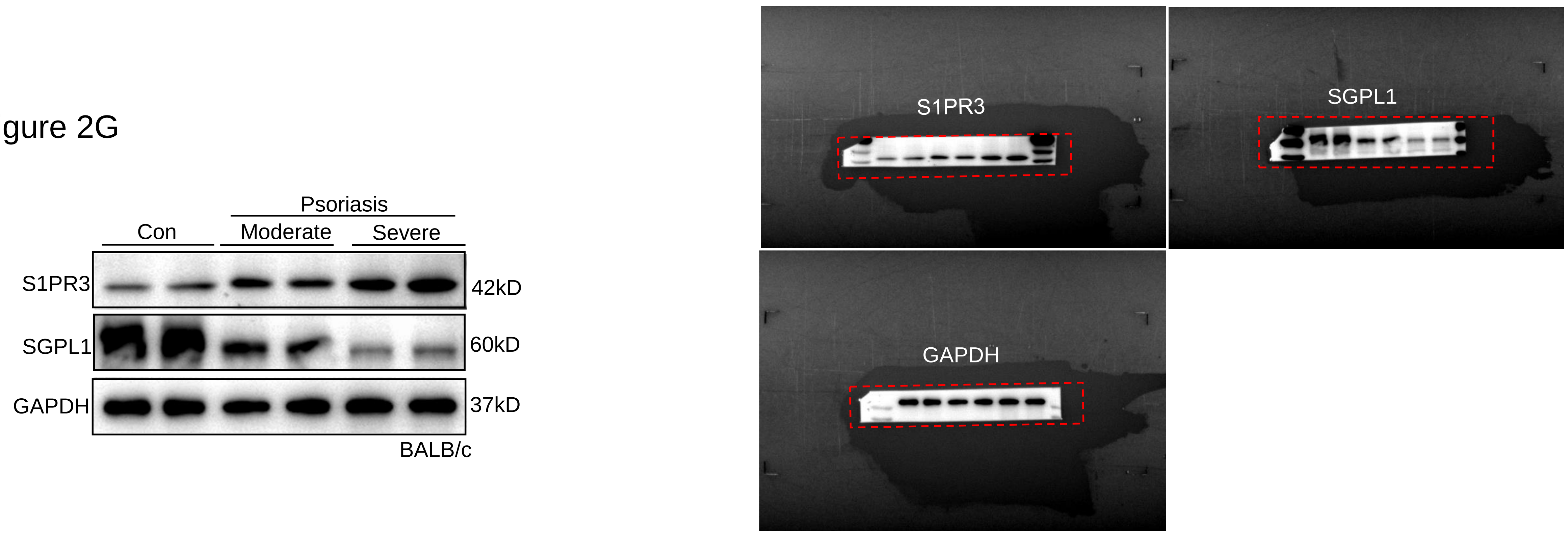

Figure 3C

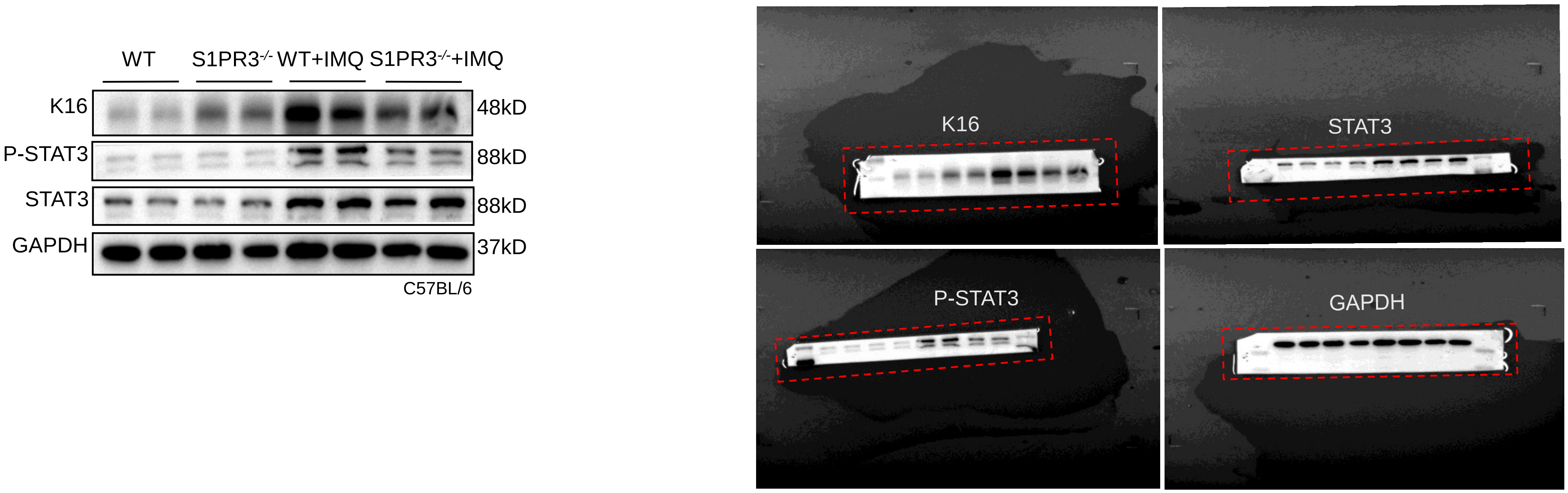

Figure 3H

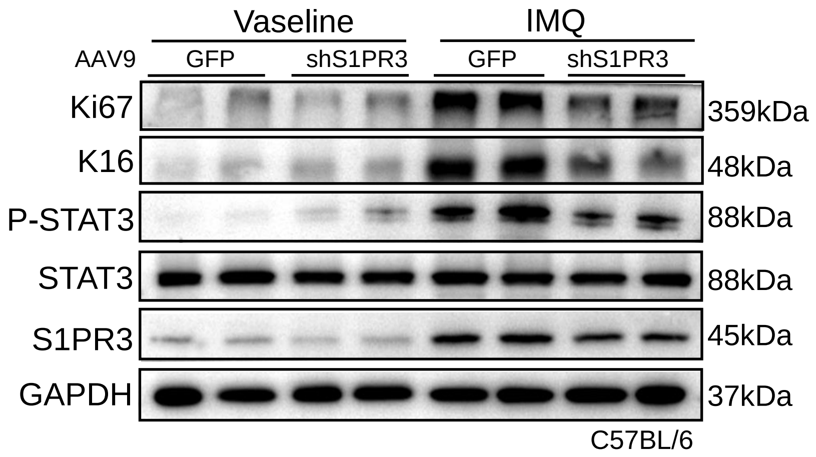
Vaseline
IMQ
AAV9
GFP
shS1PR3
GFP
shS1PR3
Ki67
K16
P-STAT3
STAT3
S1PR3
GAPDH
359kDa
48kDa
88kDa
88kDa
45kDa
37kDa
C57BL/6

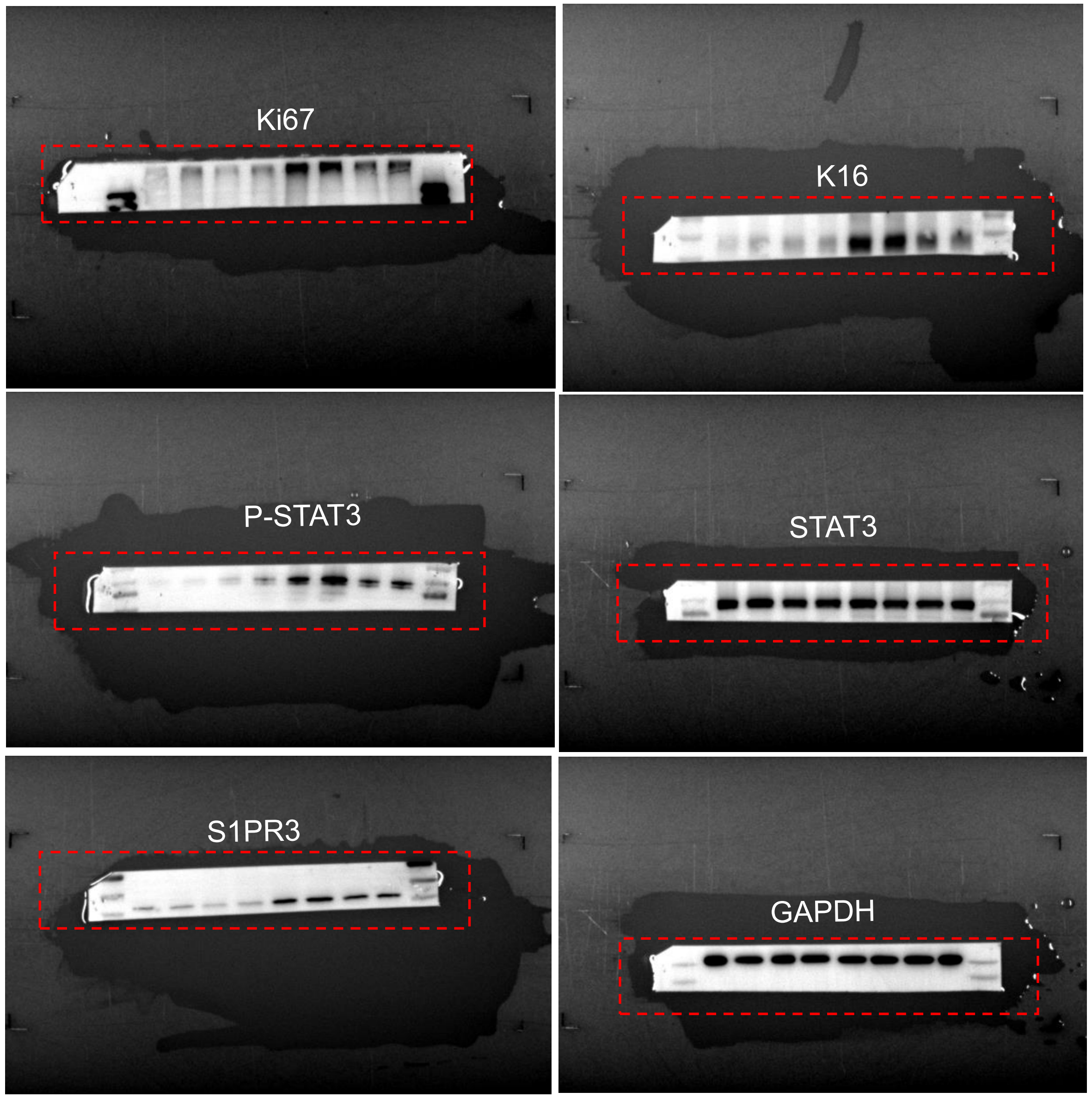
Ki67
K16
P-STAT3
STAT3
S1PR3
GAPDH

Figure 4E

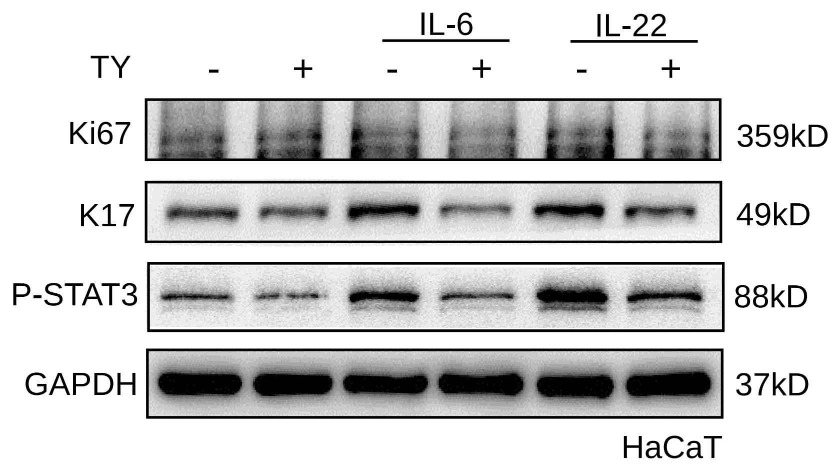

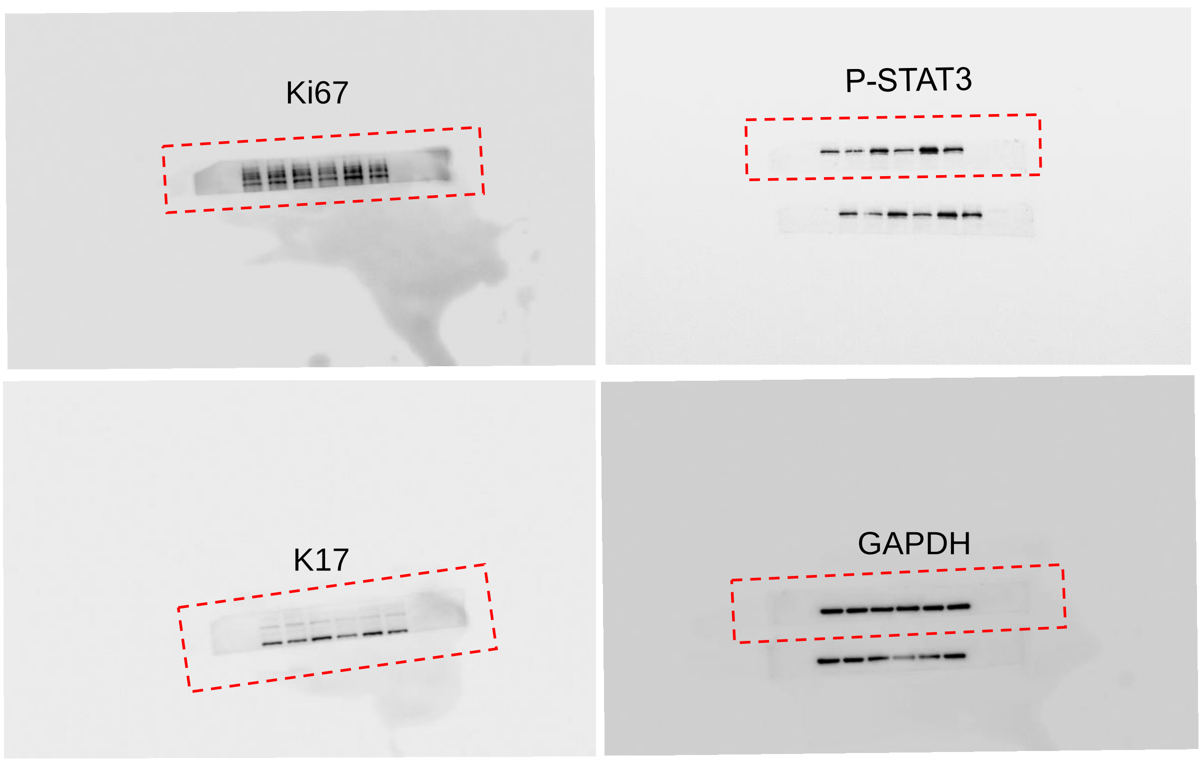

Figure 4F

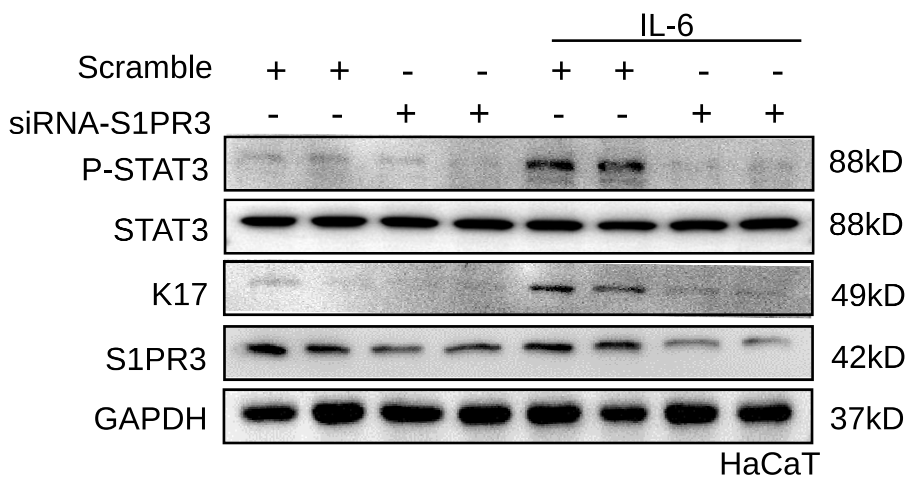

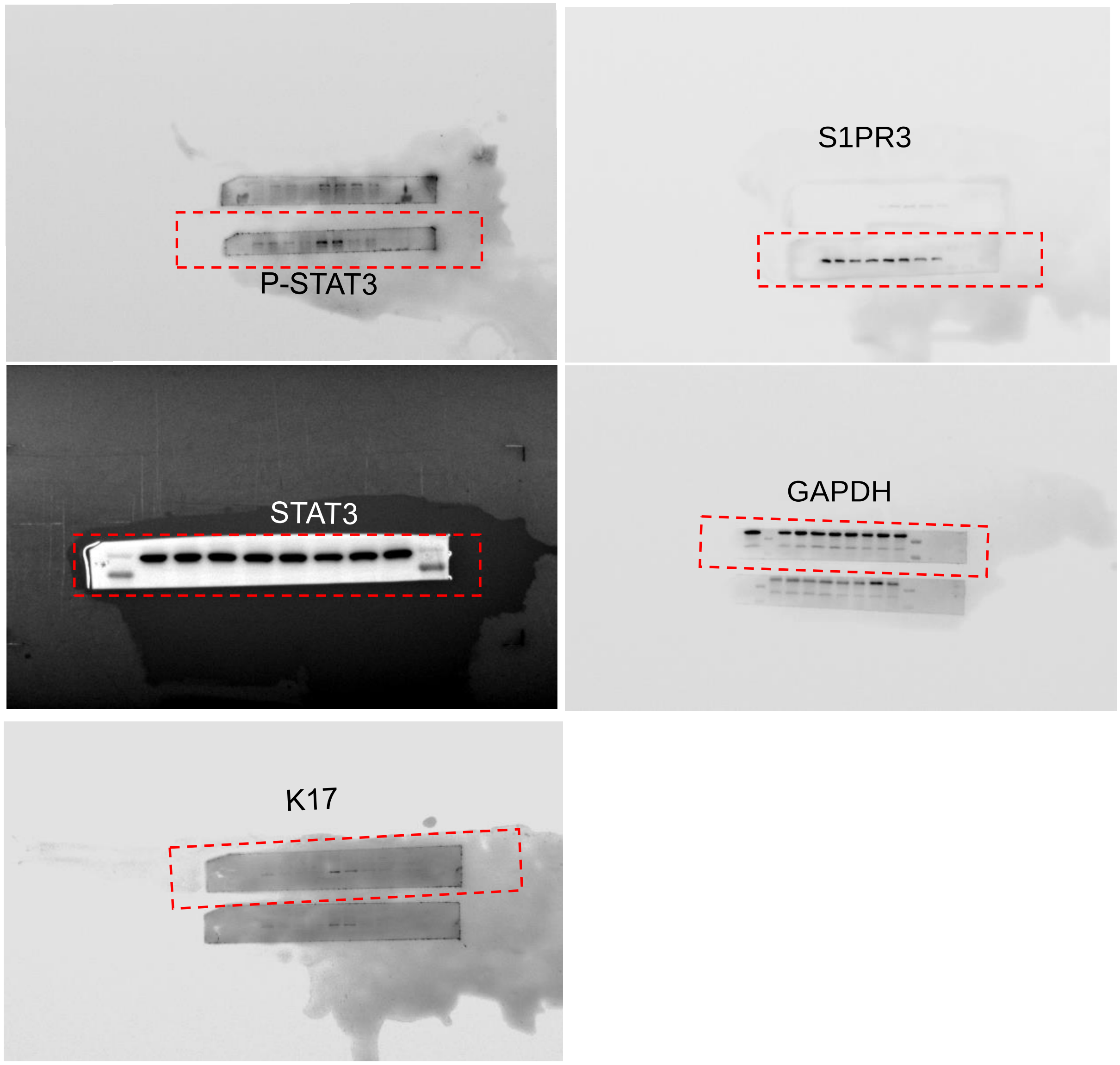

Figure 4G

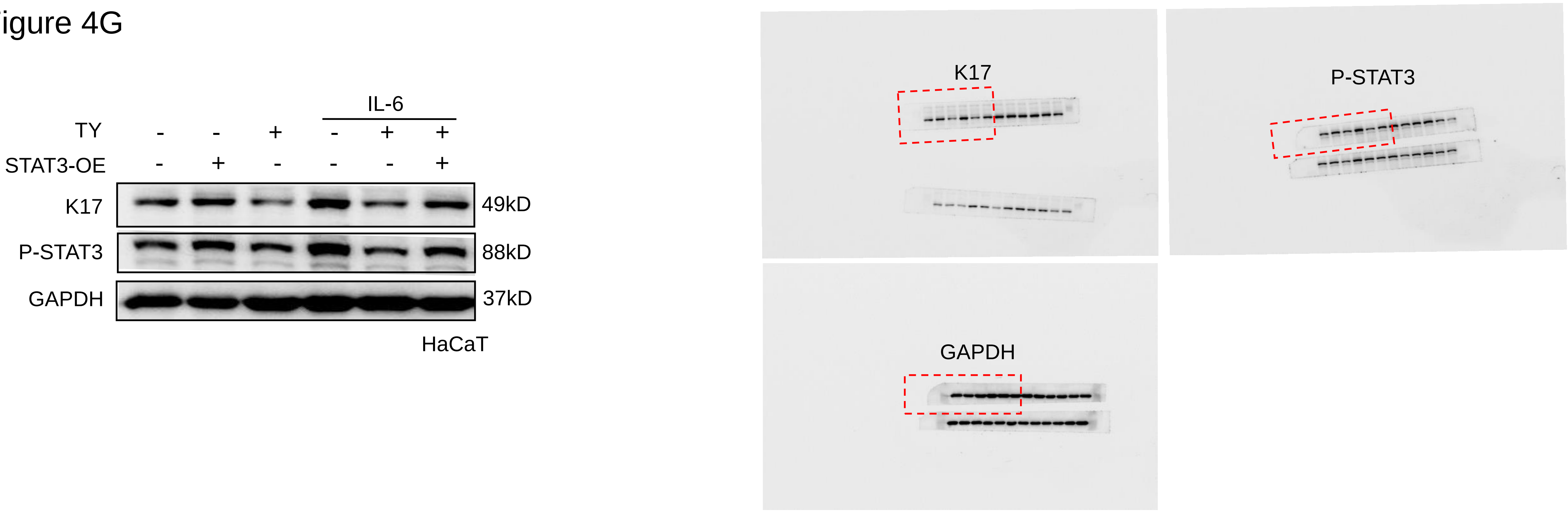

Figure 5C

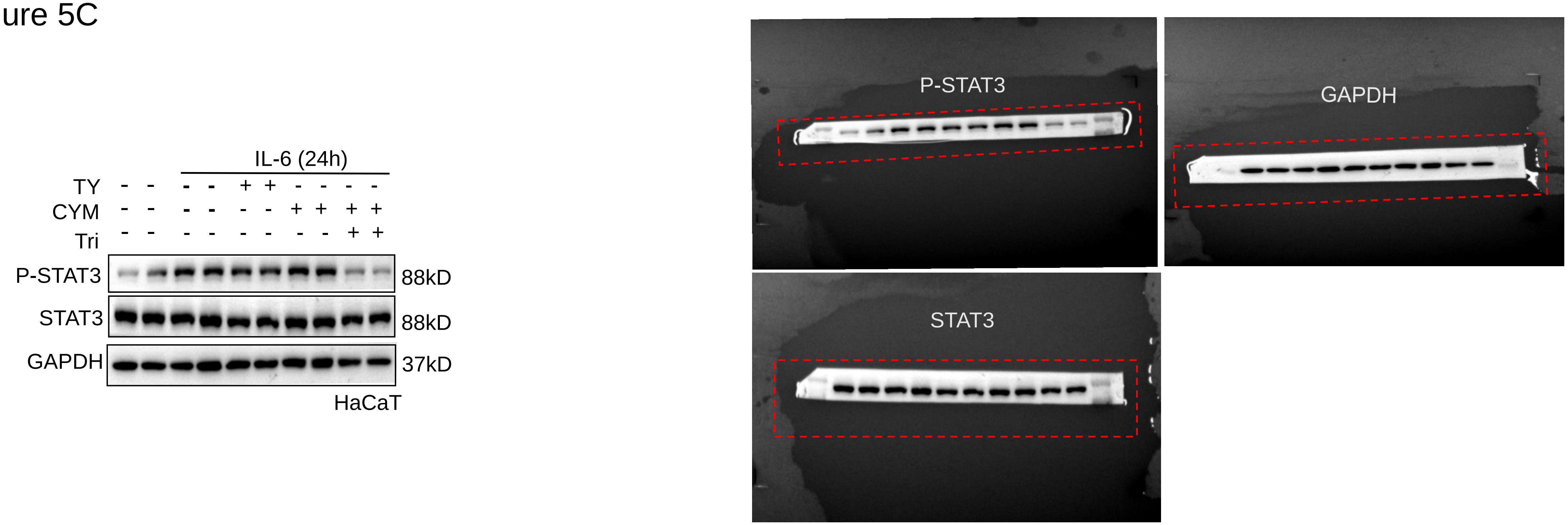

Figure 5E

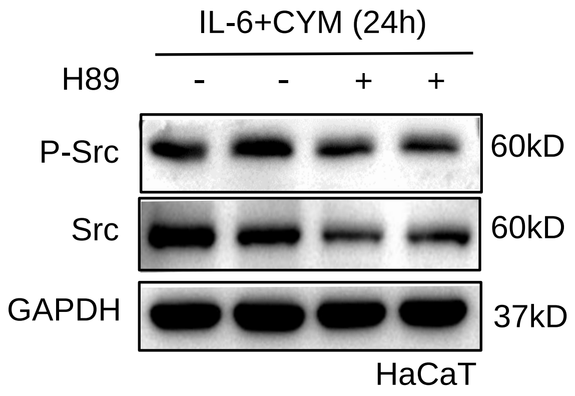

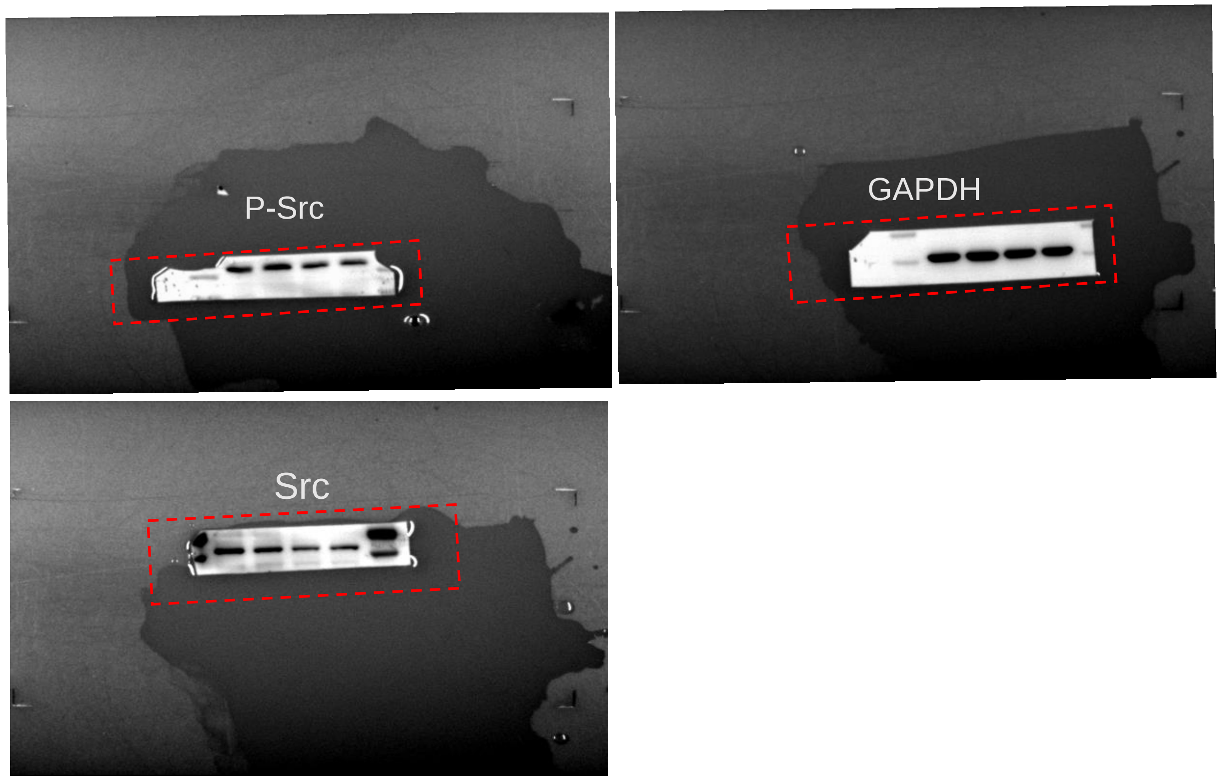

Figure 5F

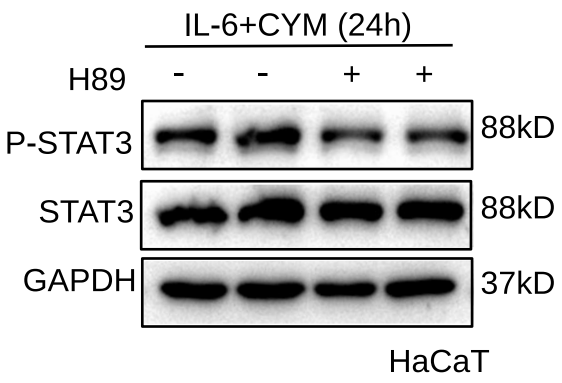

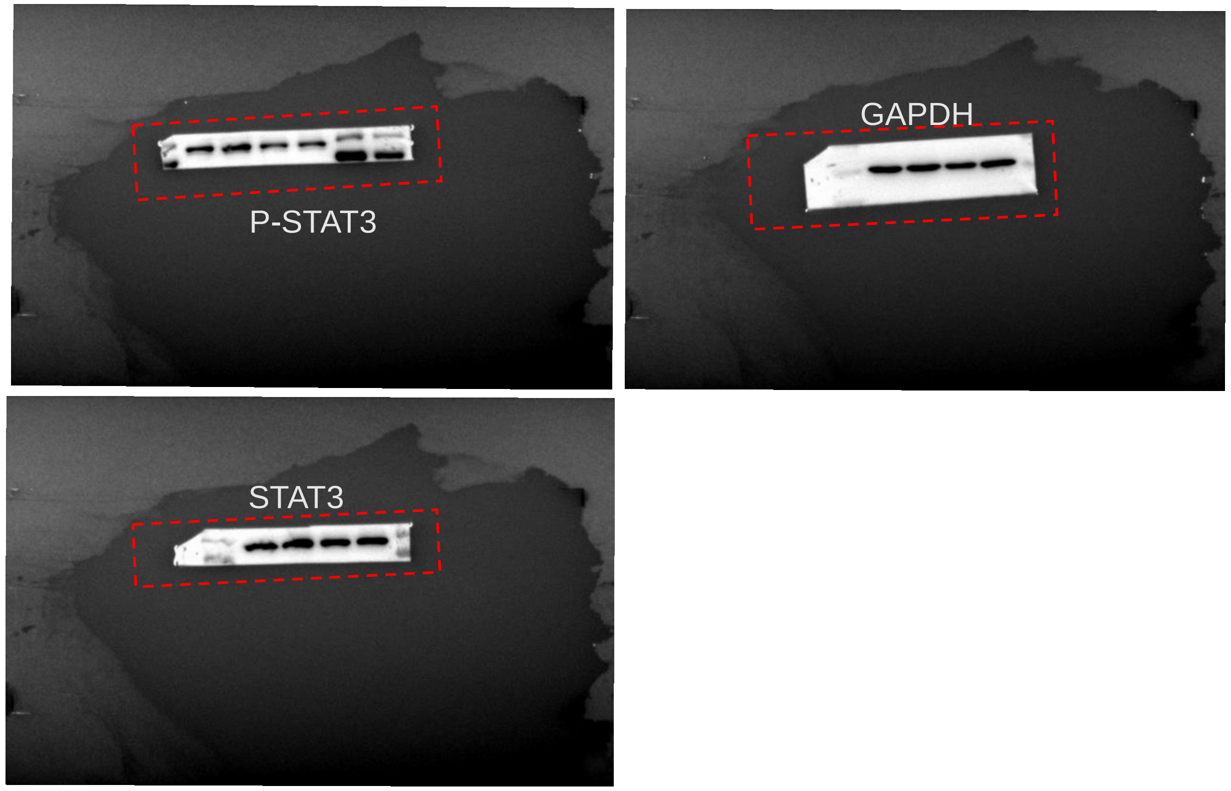

Figure 5G

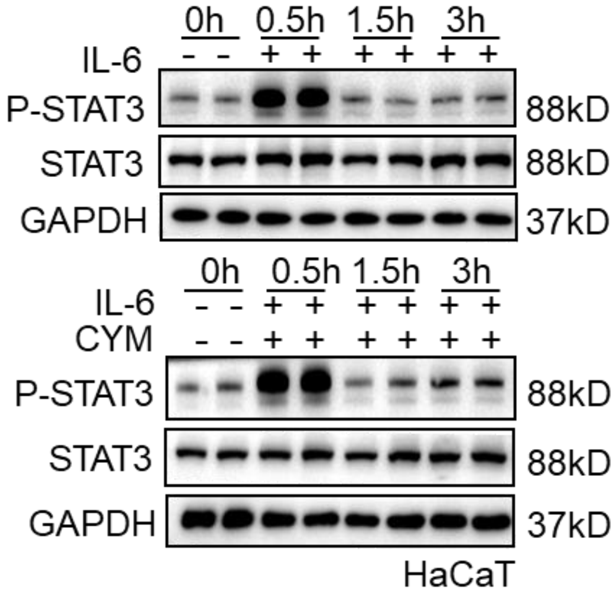

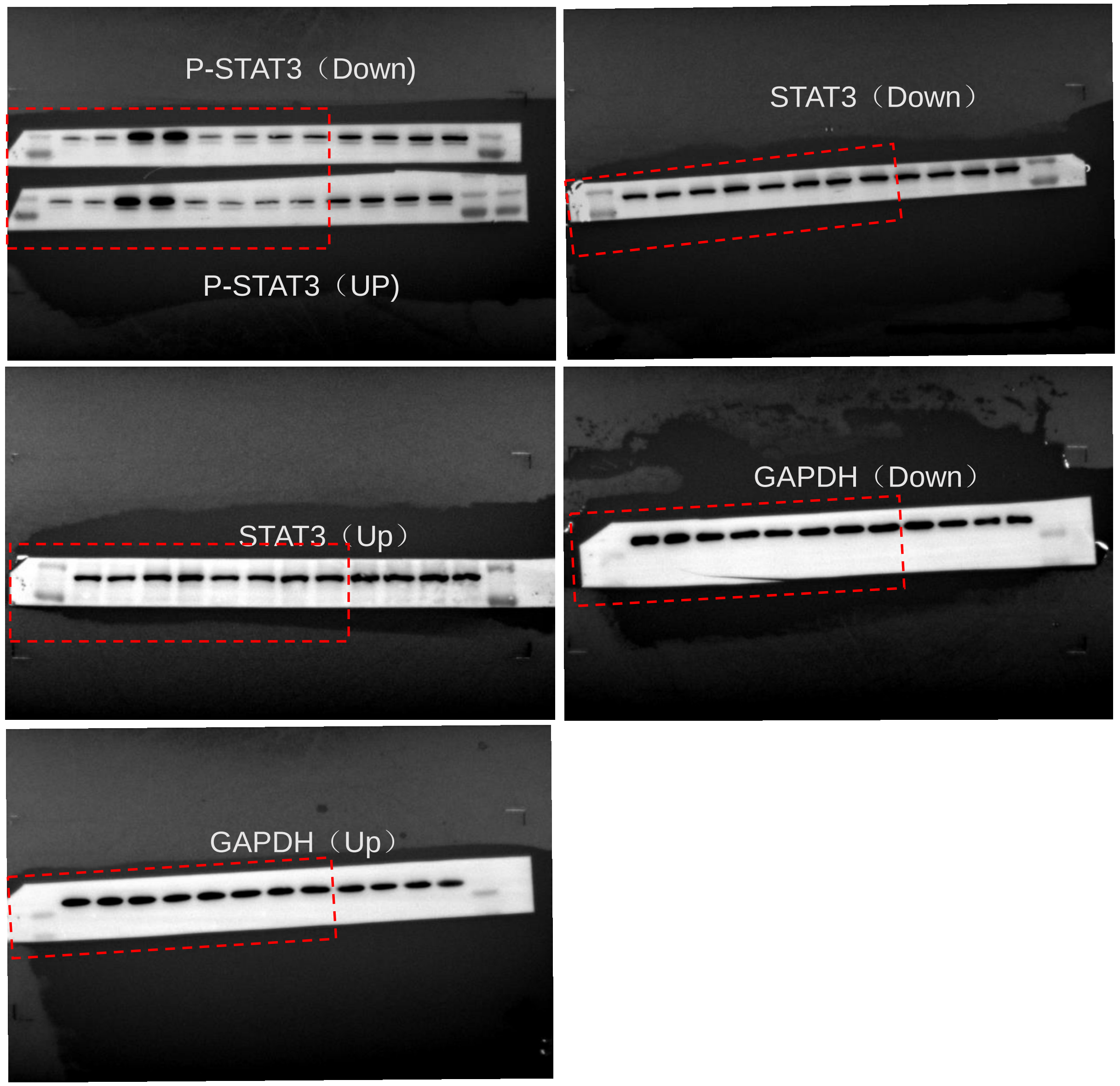

Figure 5H

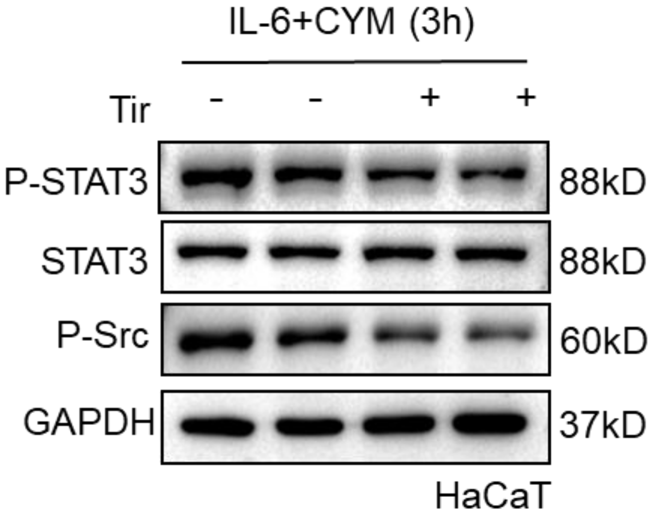

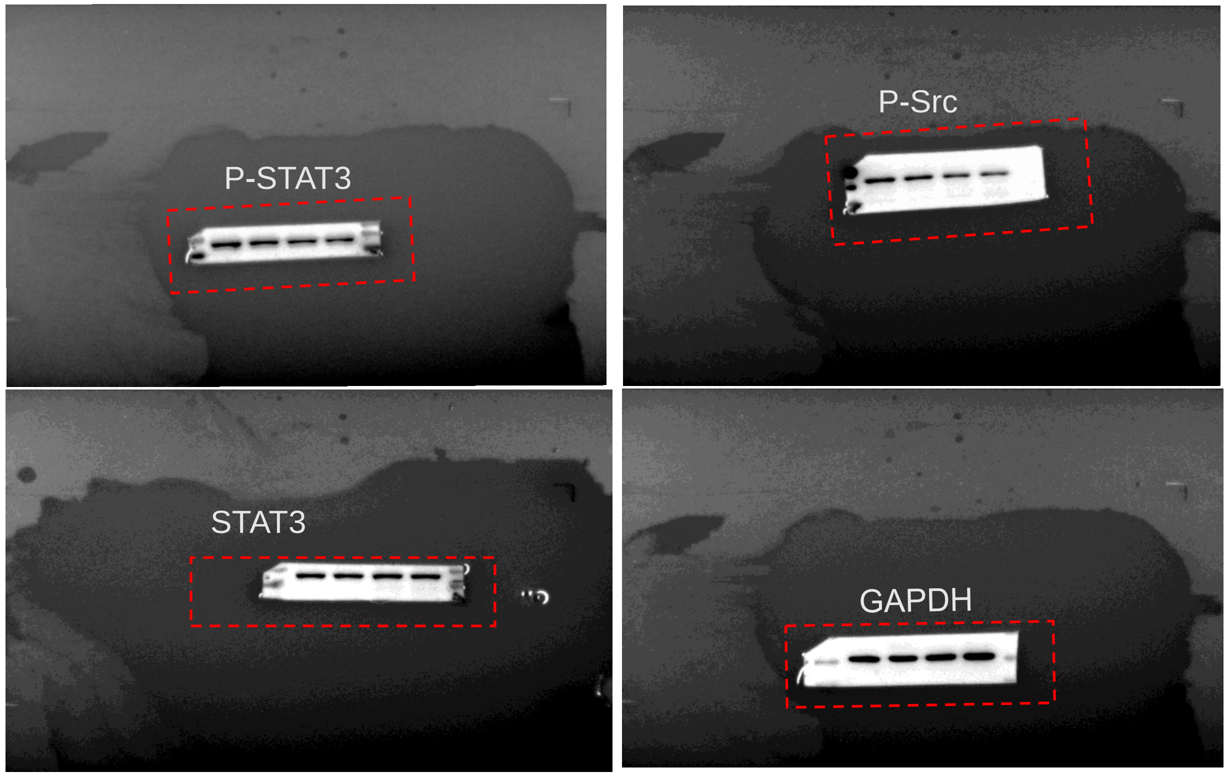

Figure 5I

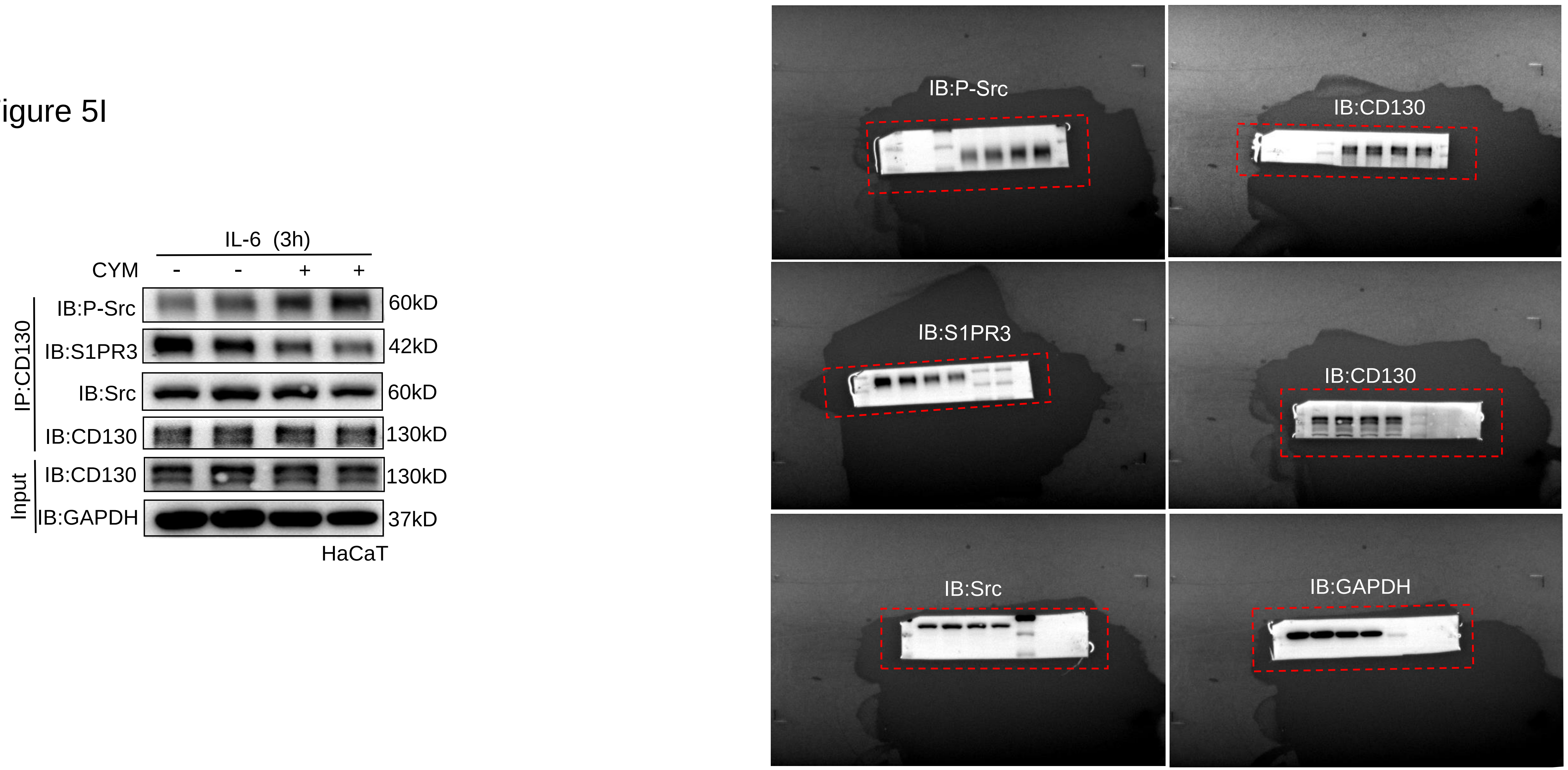

Figure 5J

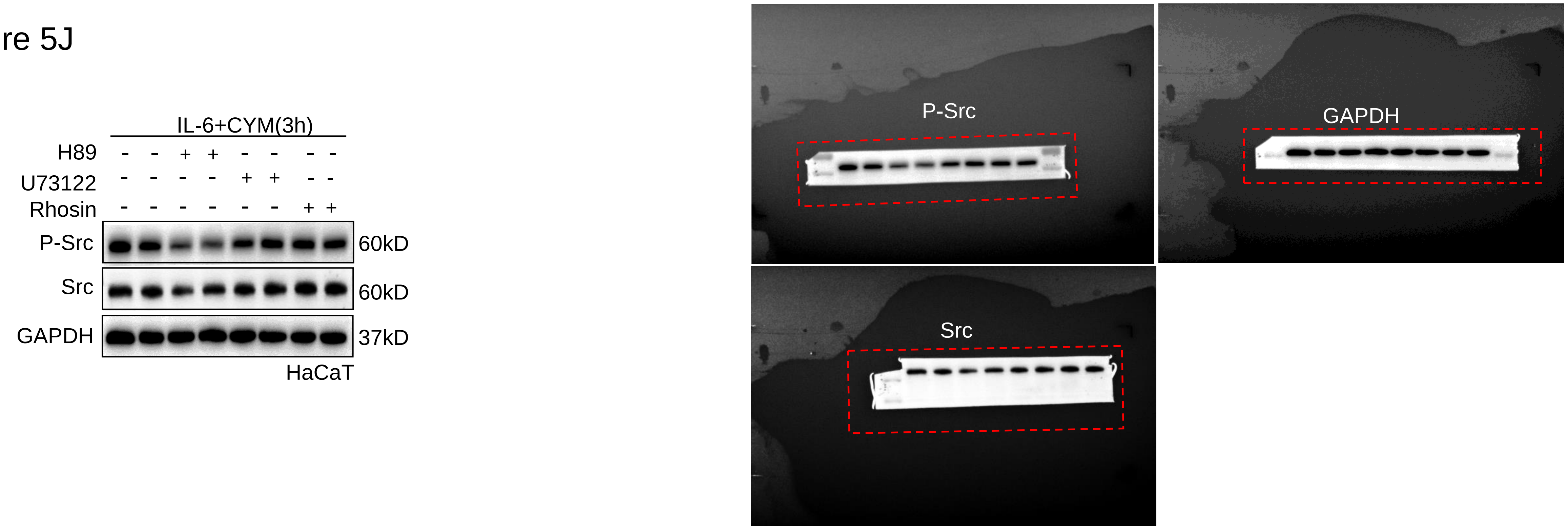

Figure 5K

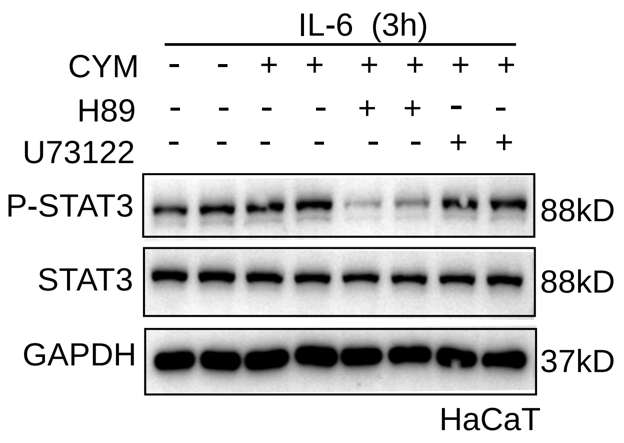

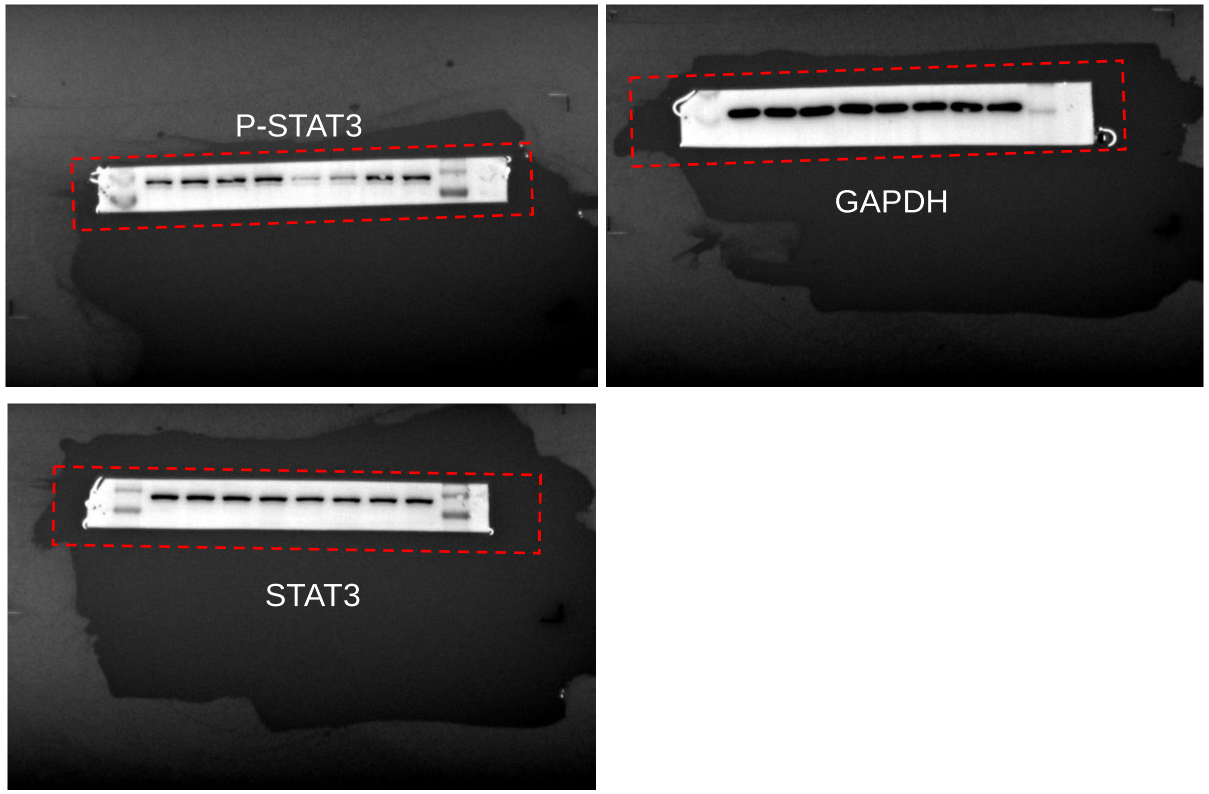

Figure 6A

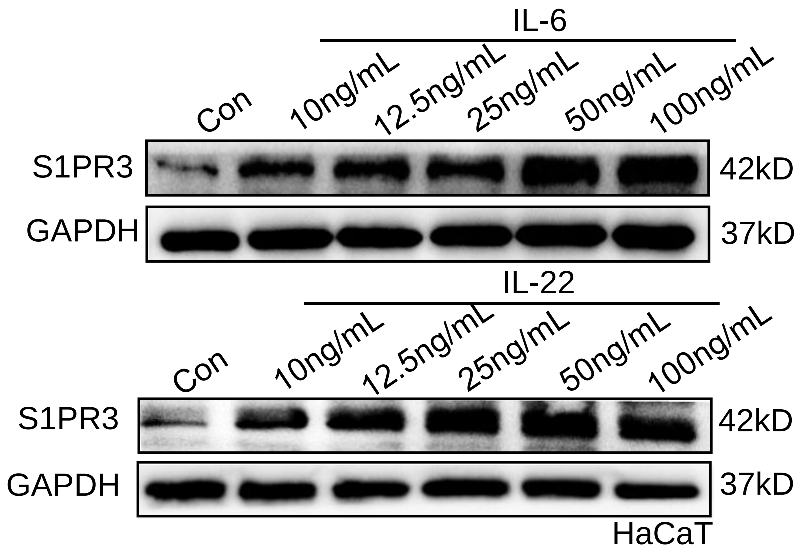

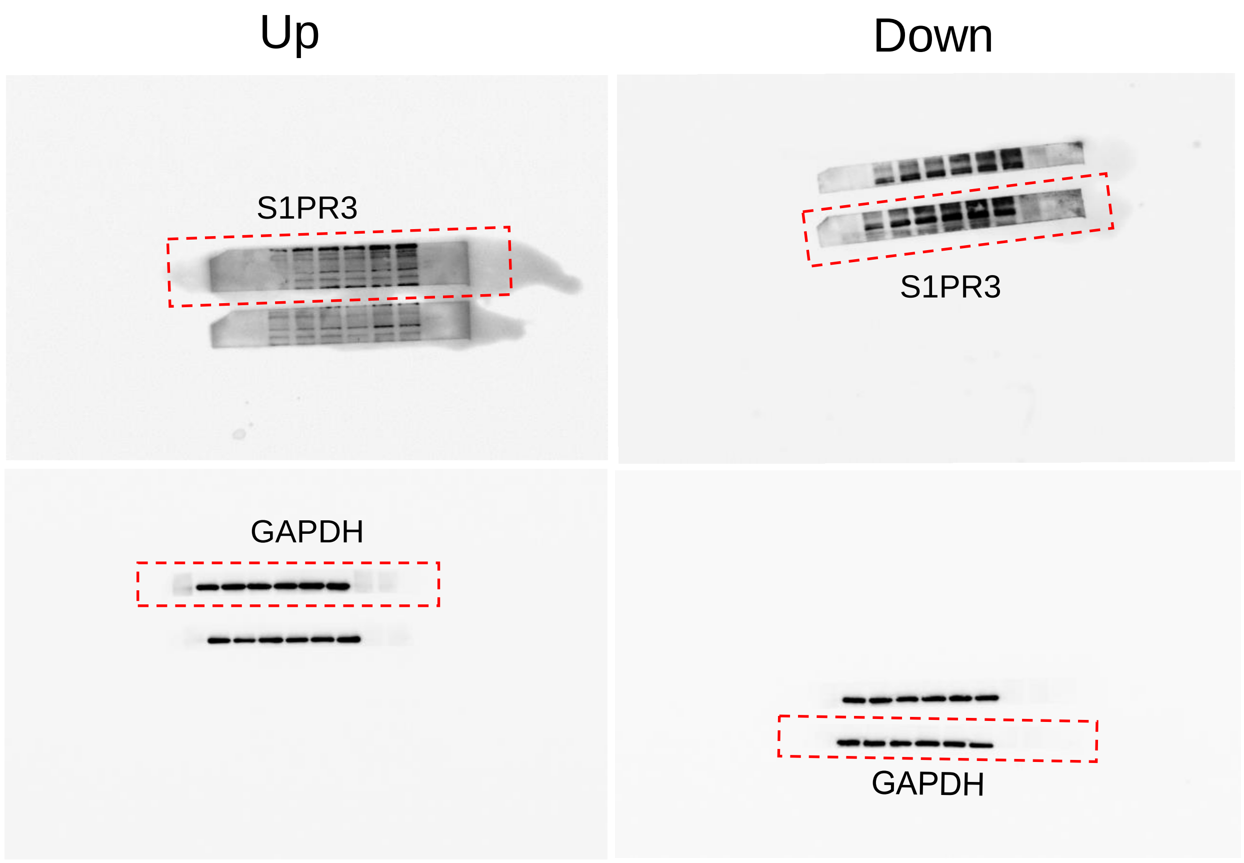

Figure 6B

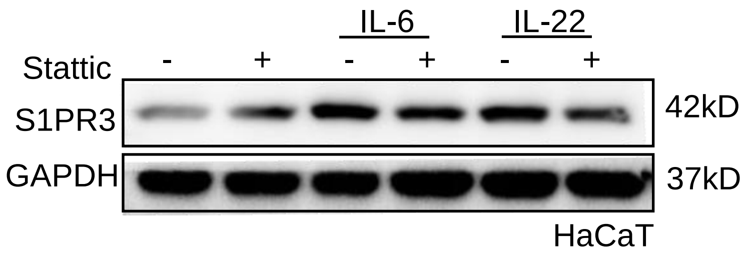

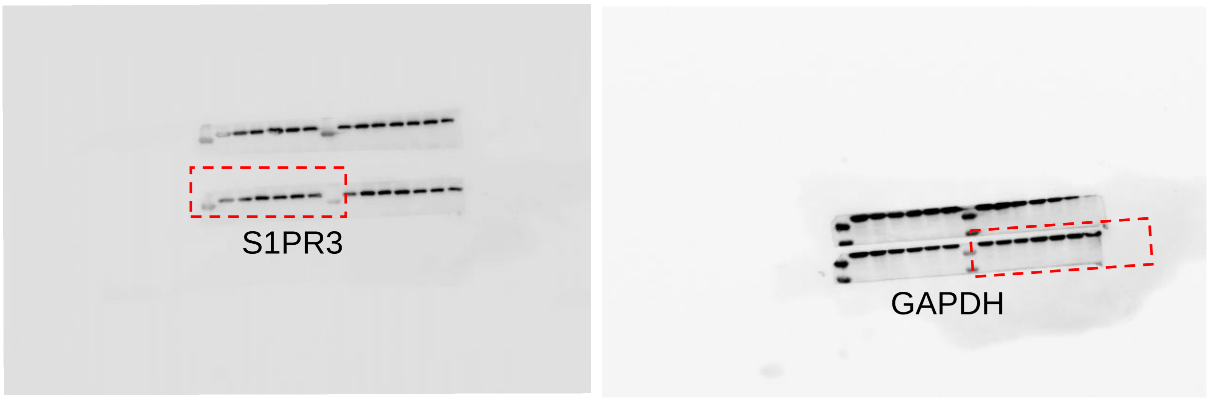

Figure 6C

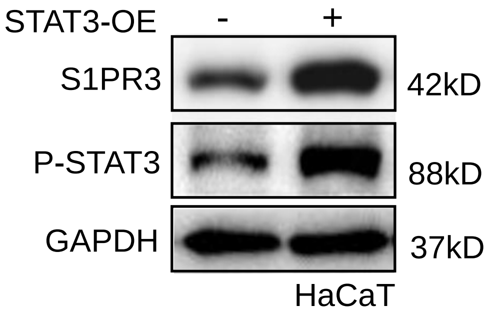

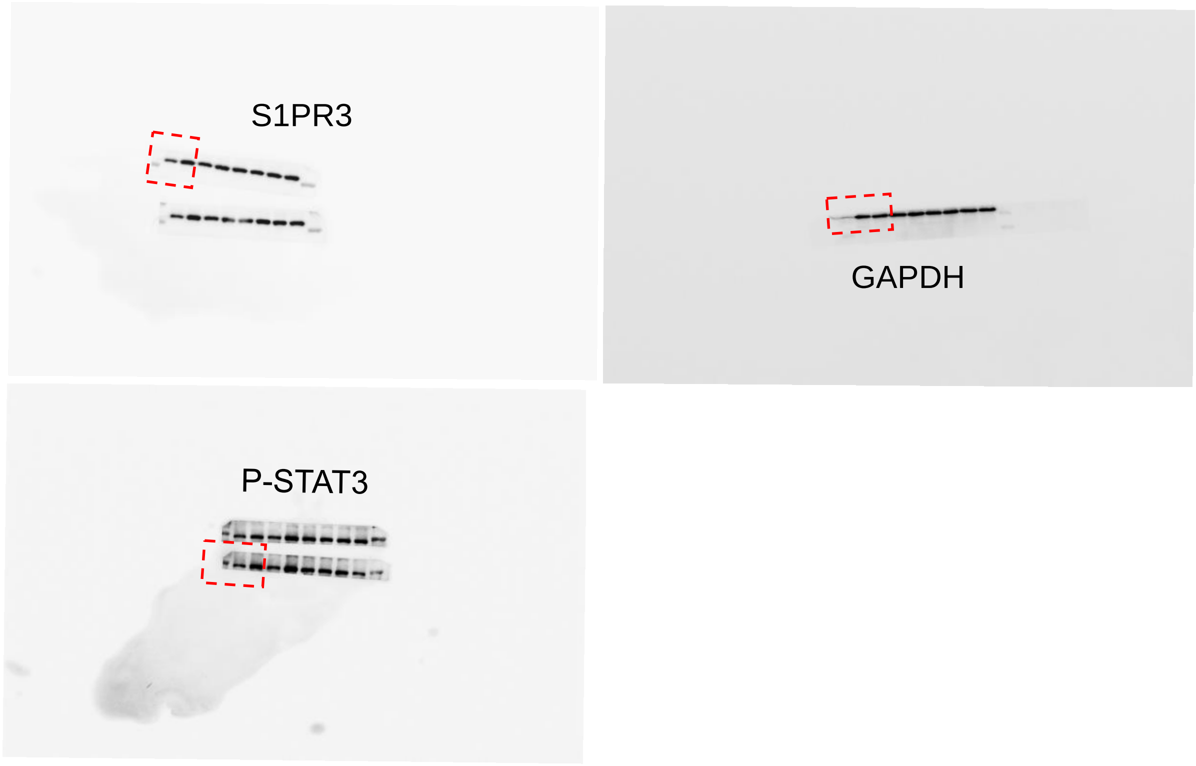

Figure 6E

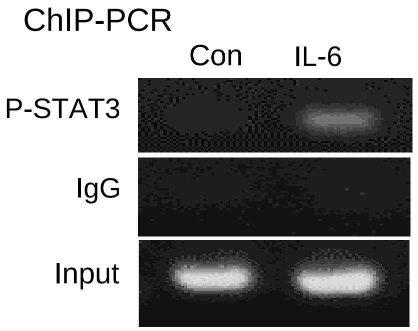

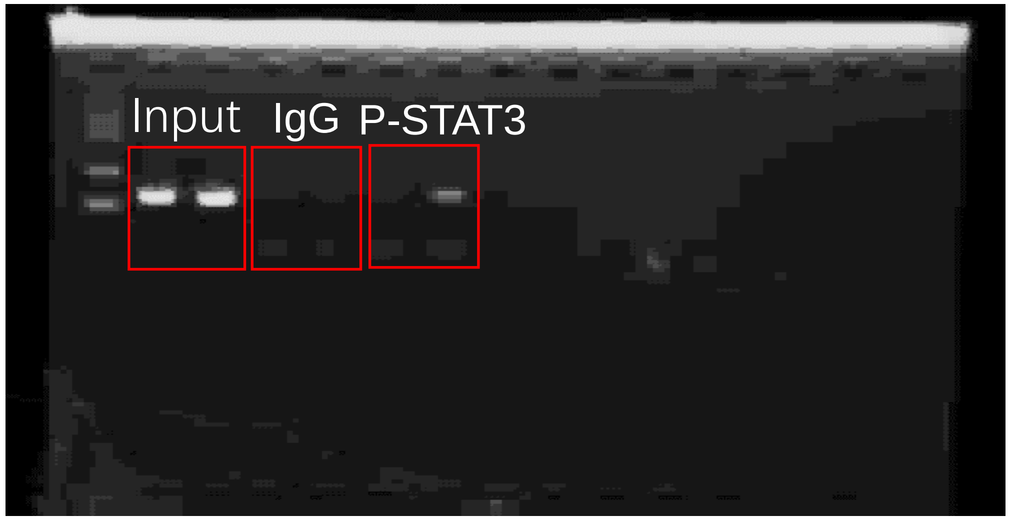

Figure 7D

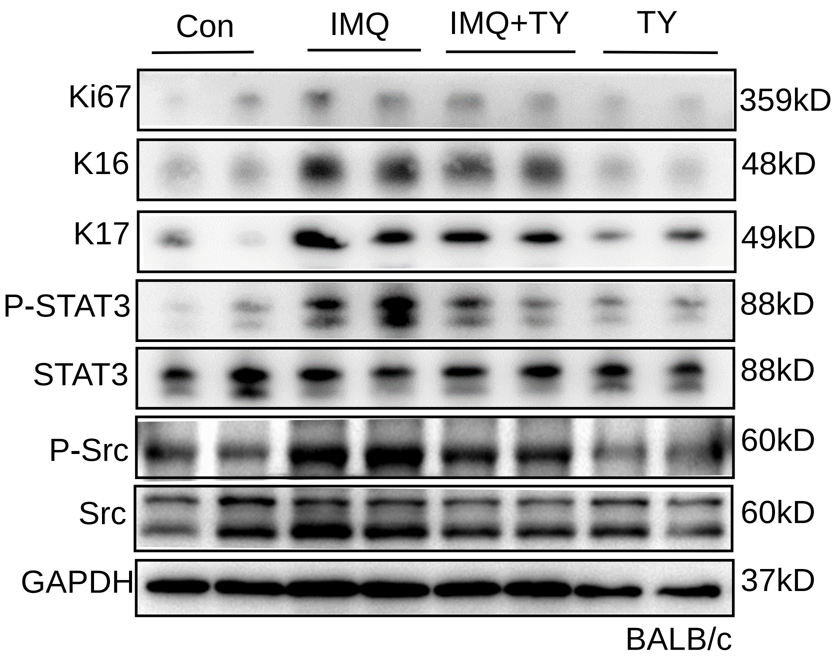

Con
IMQ
IMQ+TY
TY
Ki67
359kD
K16
48kD
K17
49kD
P-STAT3
88kD
STAT3
88kD
P-Src
60kD
Src
60kD
GAPDH
37kD
BALB/c

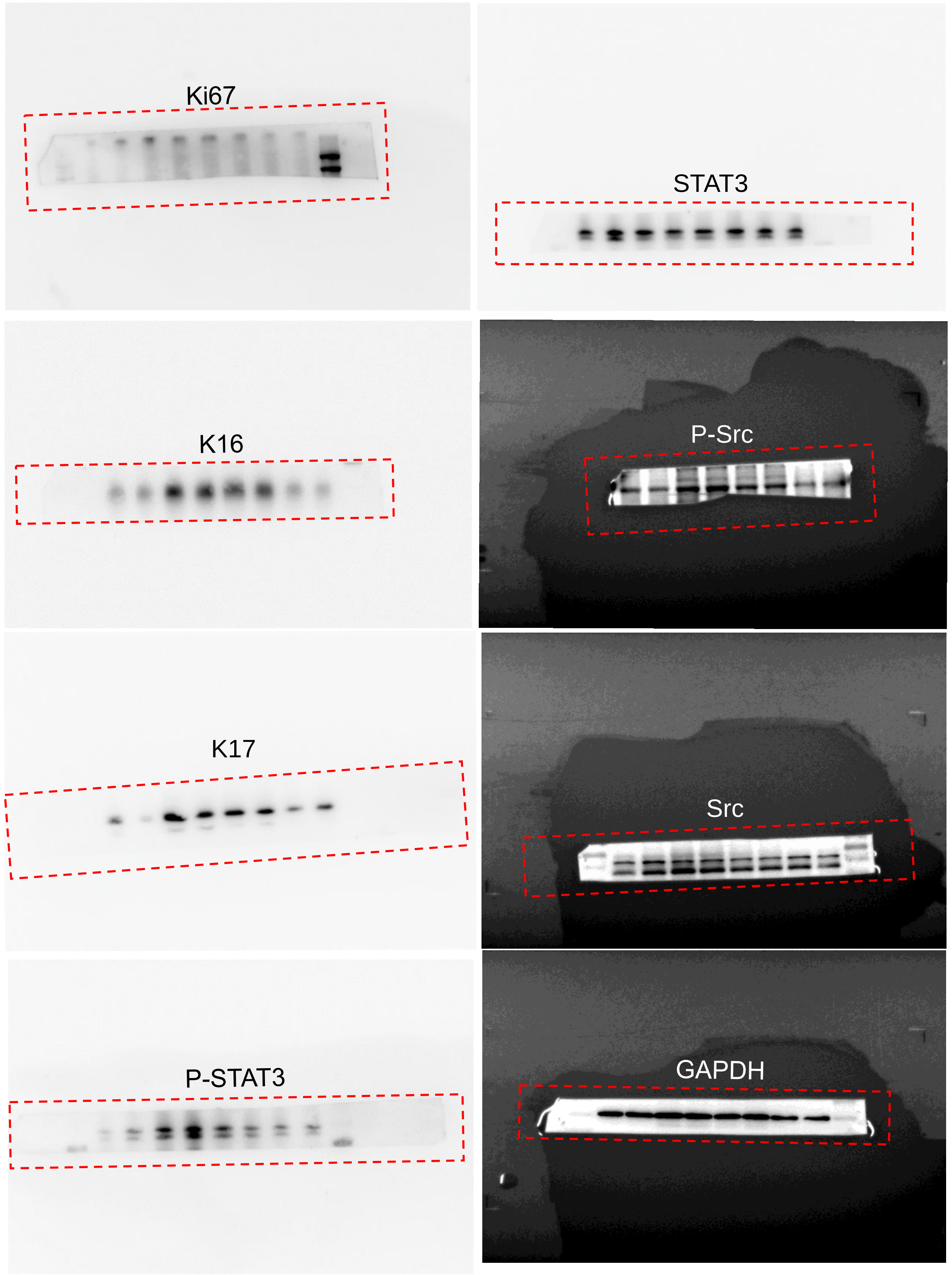

Ki67
STAT3
K16
P-Src
K17
Src
P-STAT3
GAPDH

Supplementary Figure 1F

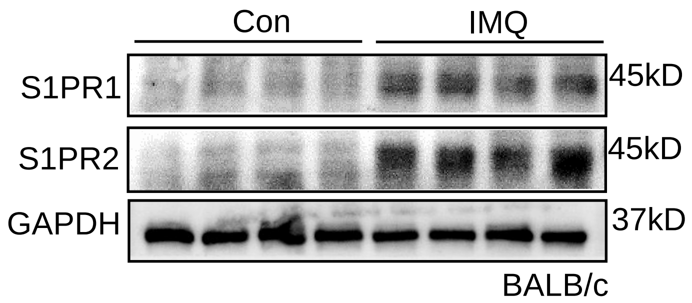

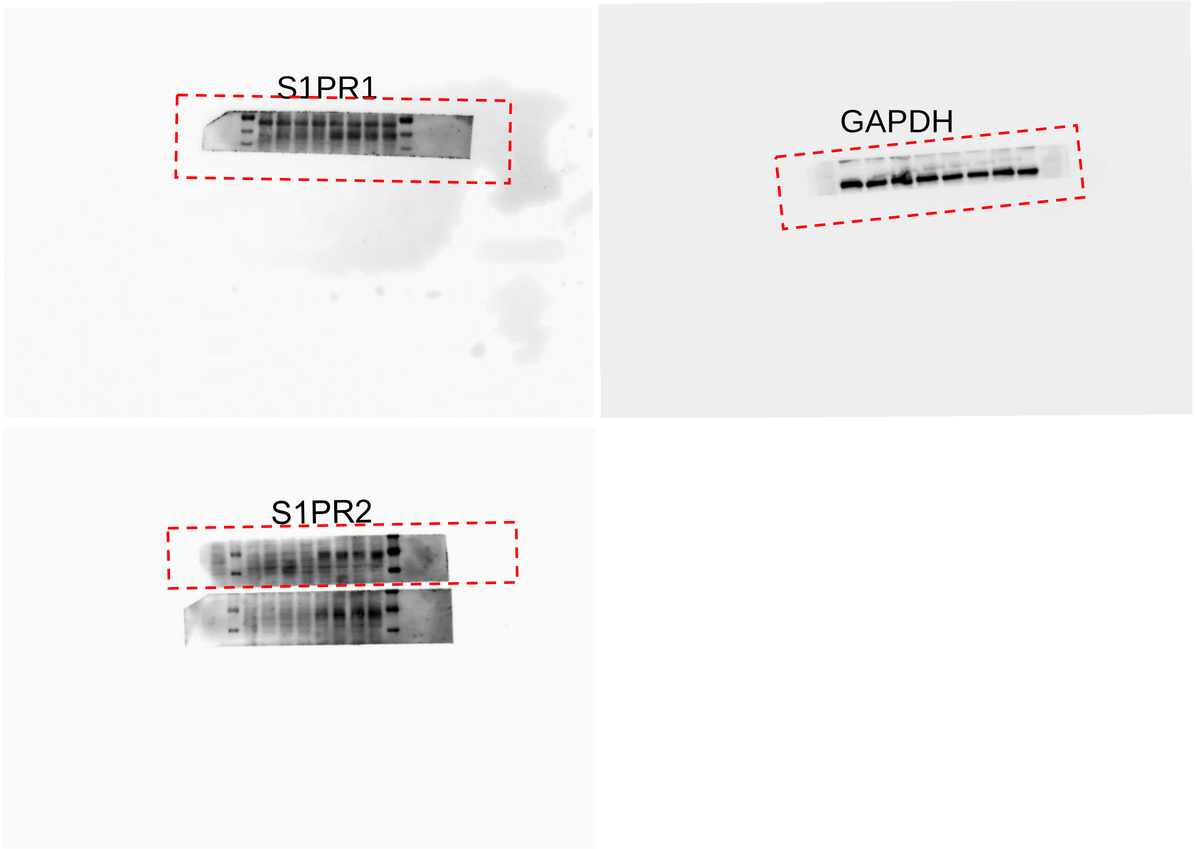

Supplementary Figure 3A

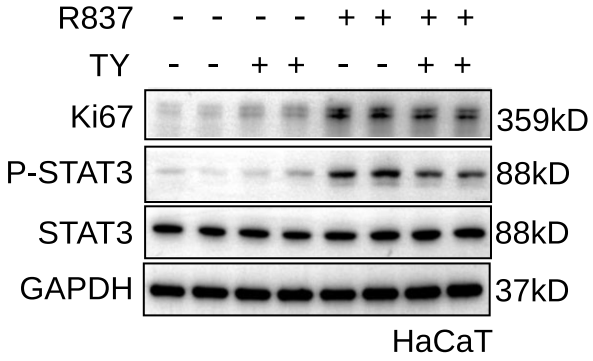

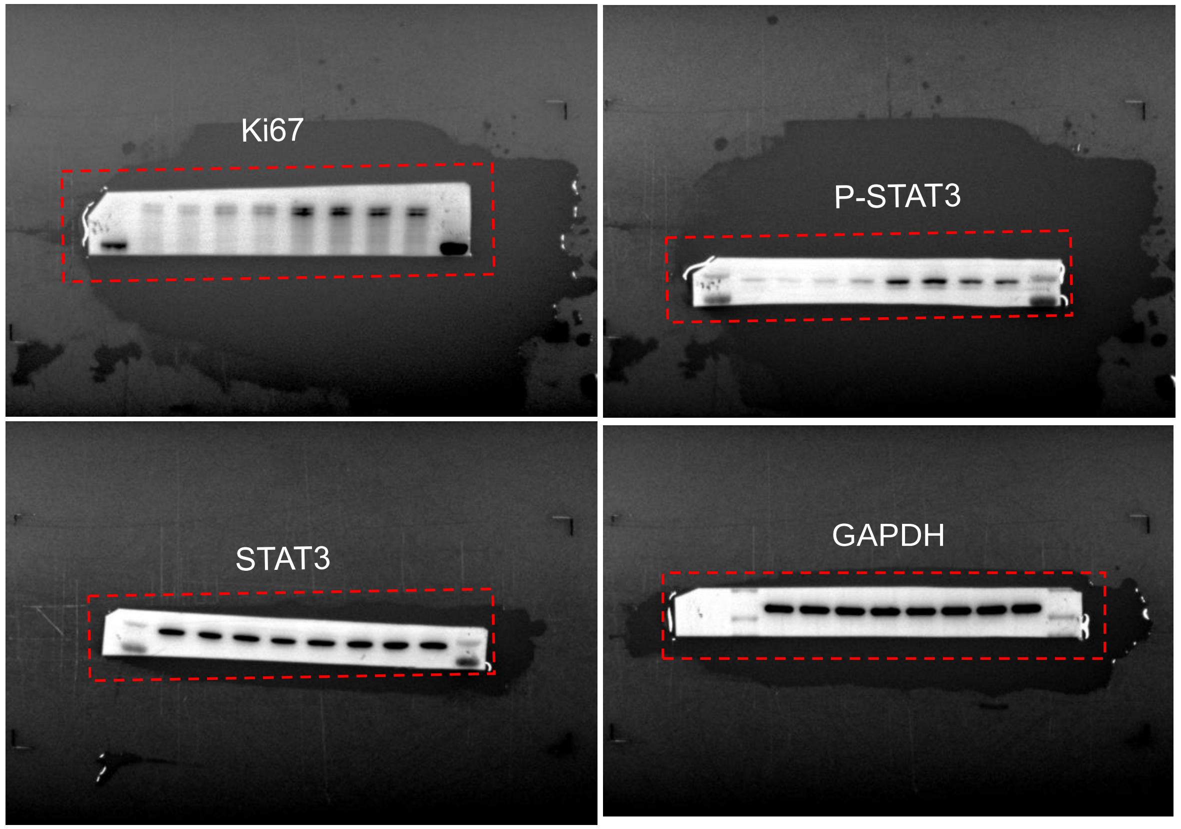

Supplementary Figure 3C

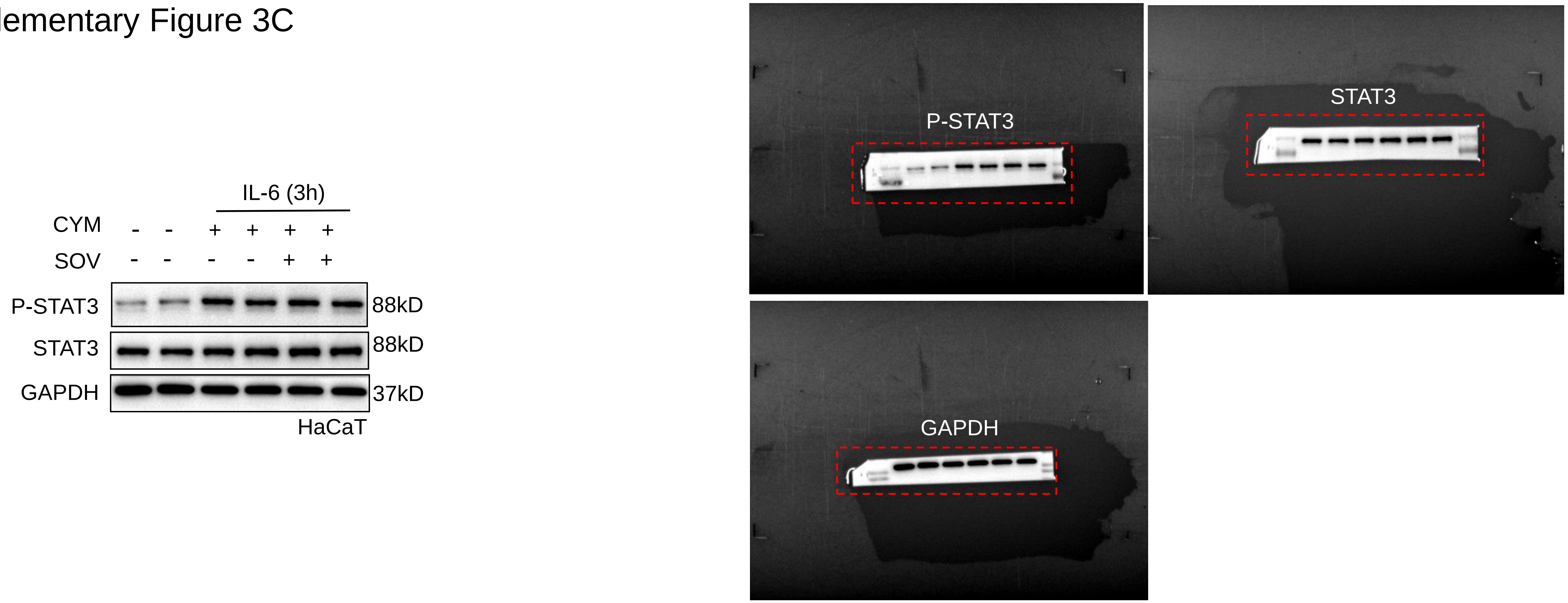

Supplementary Figure 3D

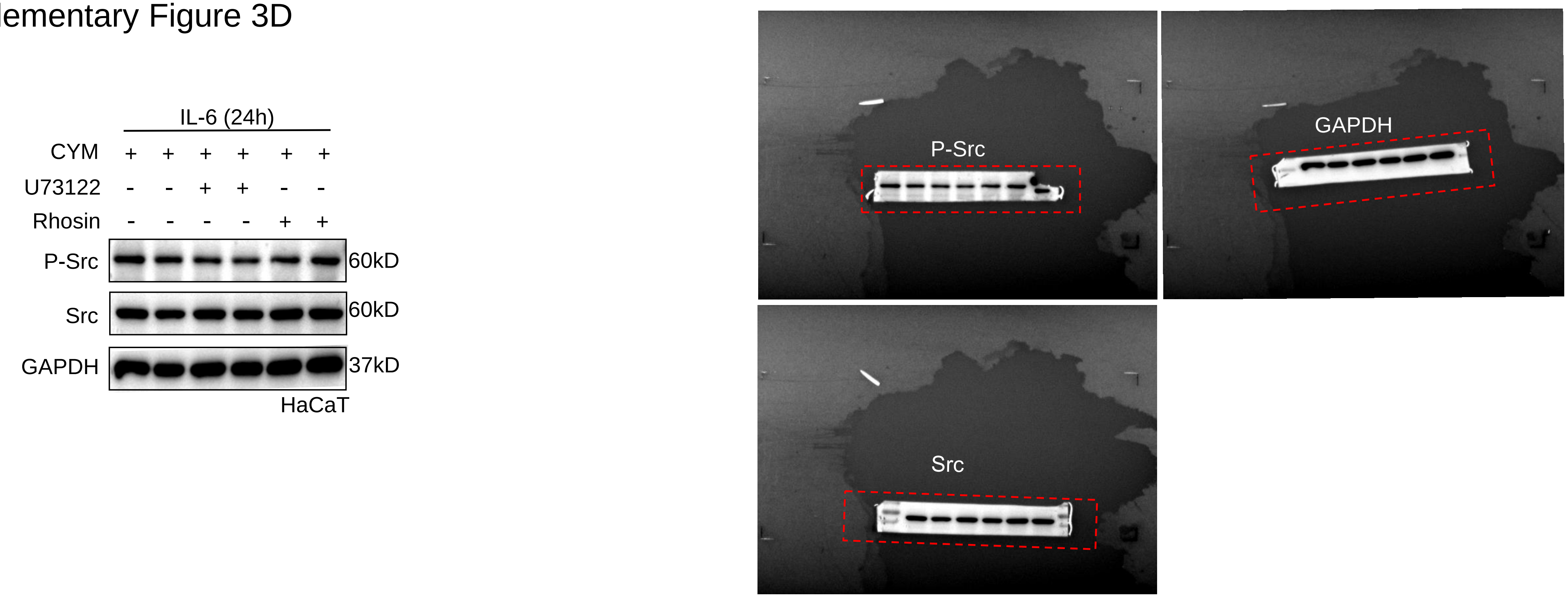

Supplementary Figure 3E

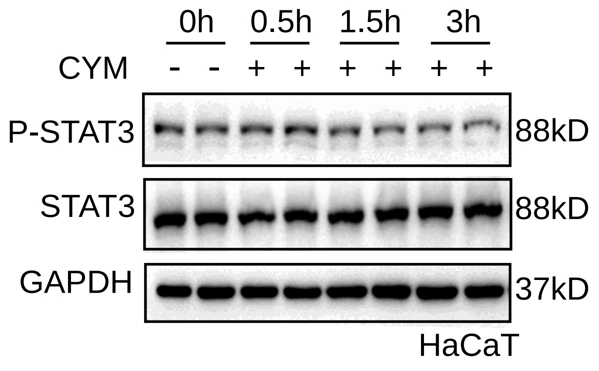

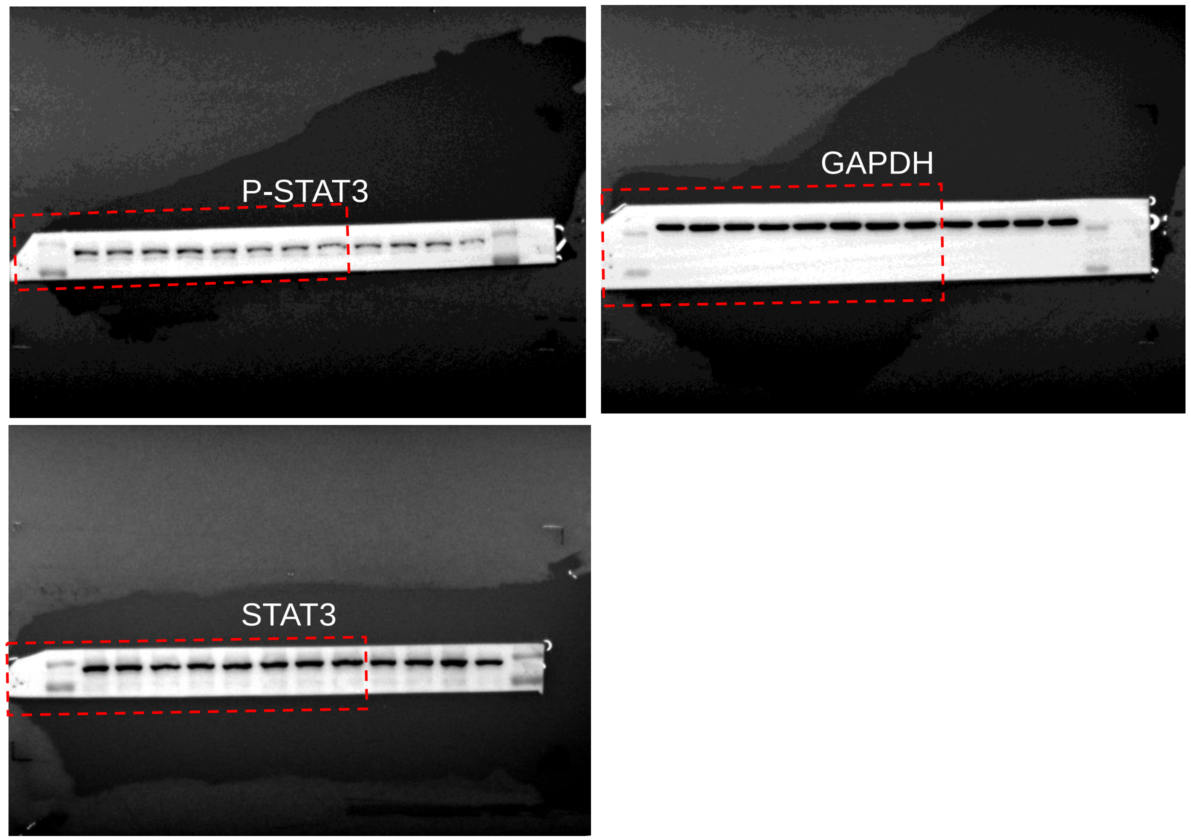

Supplementary Figure 3F

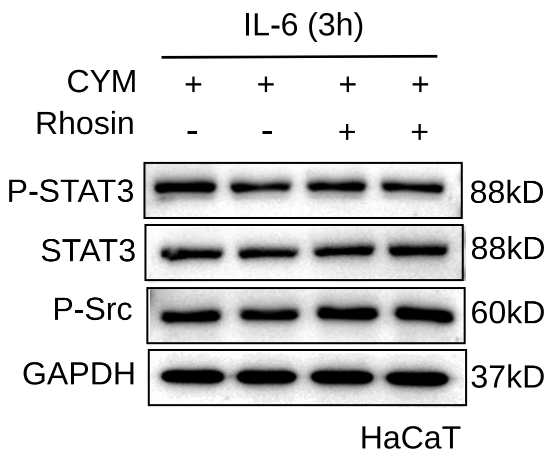

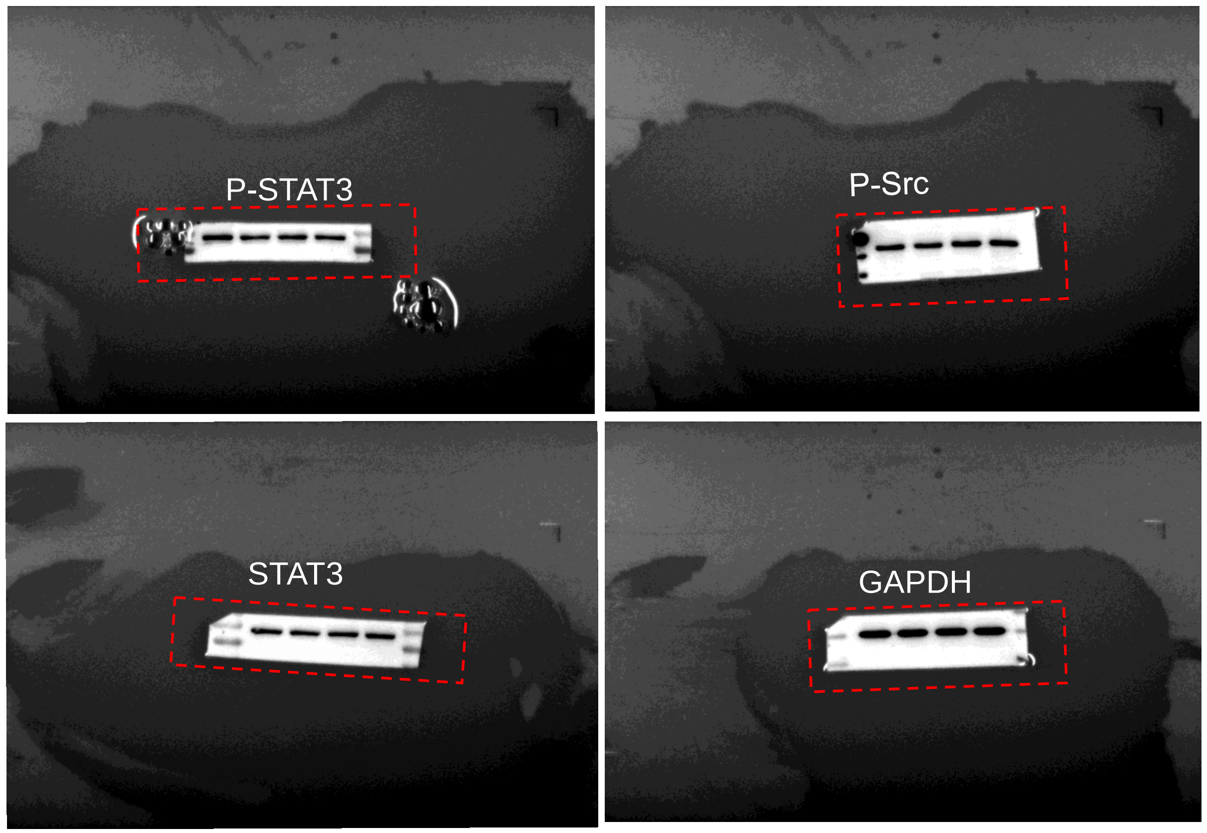

Supplementary Figure 3J

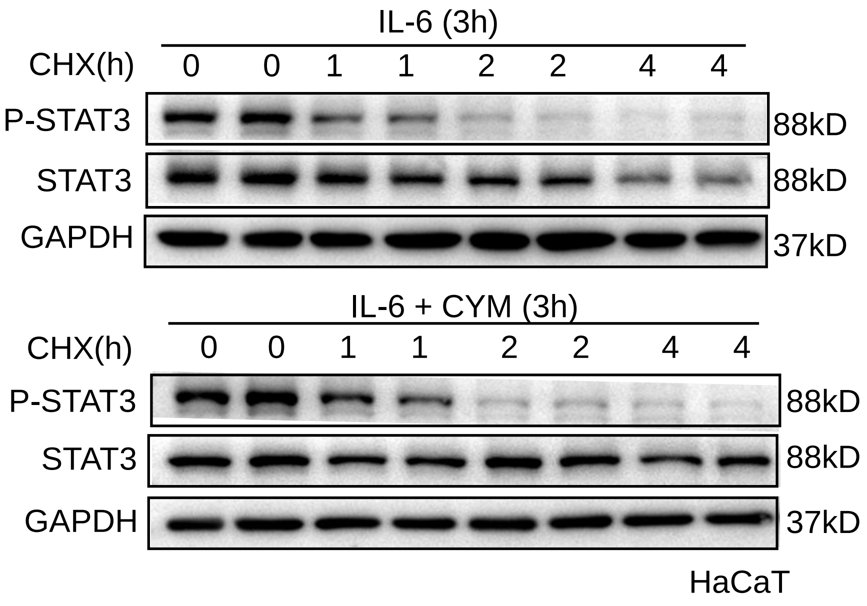

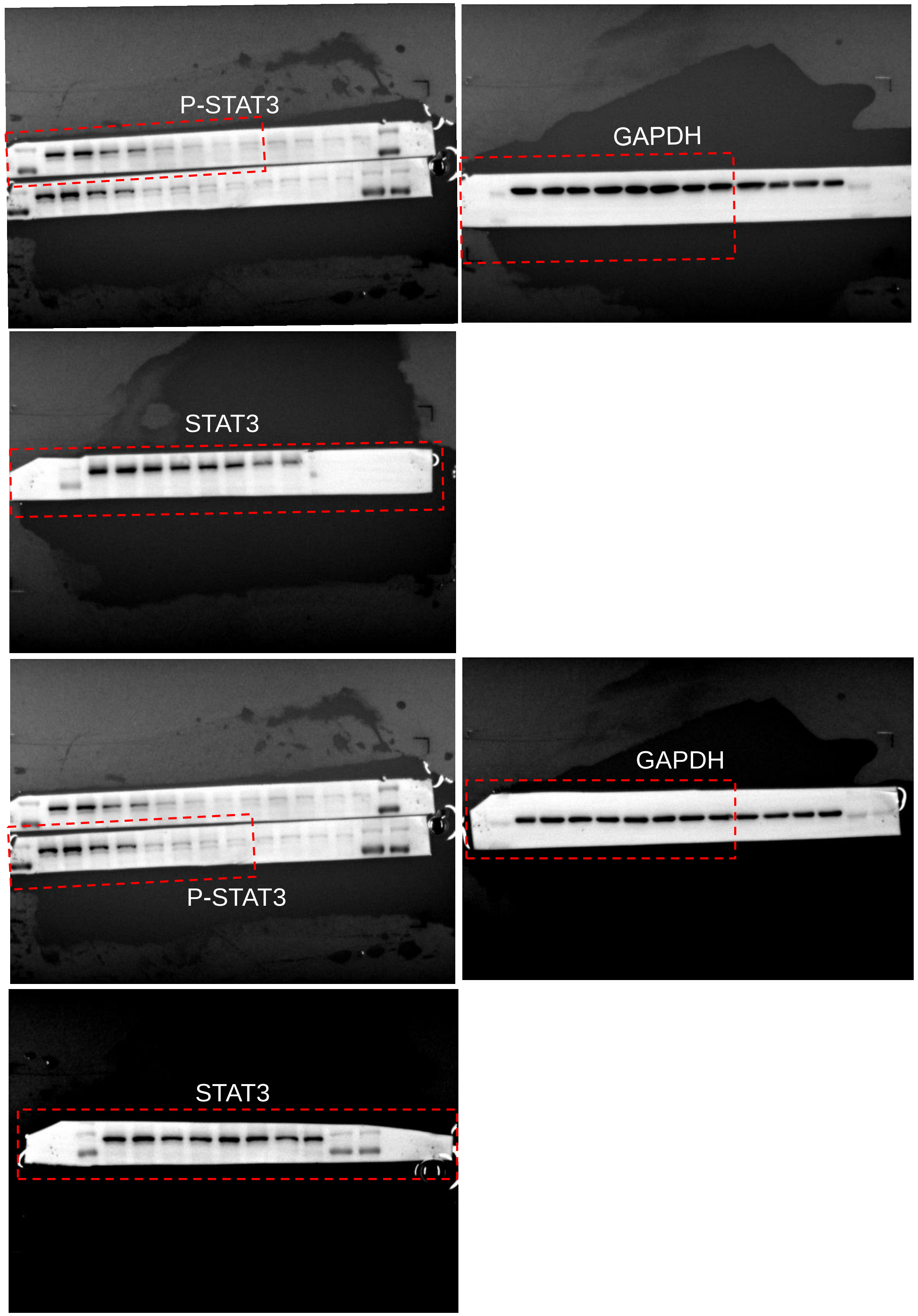

Supplementary Figure 3K

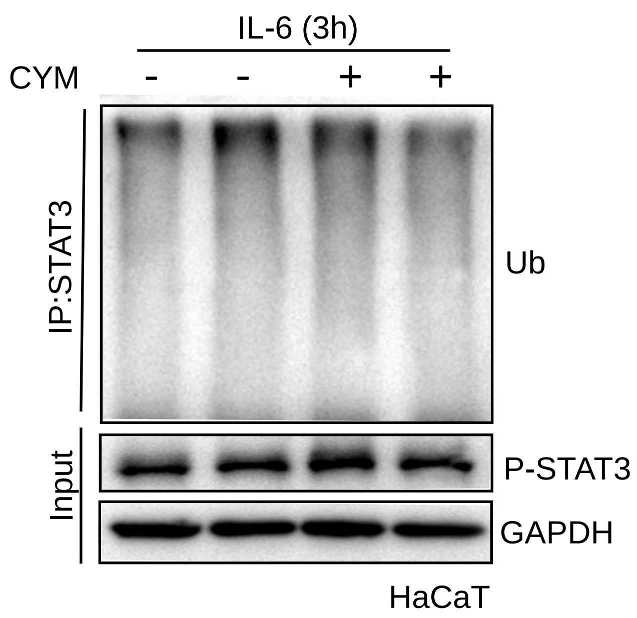

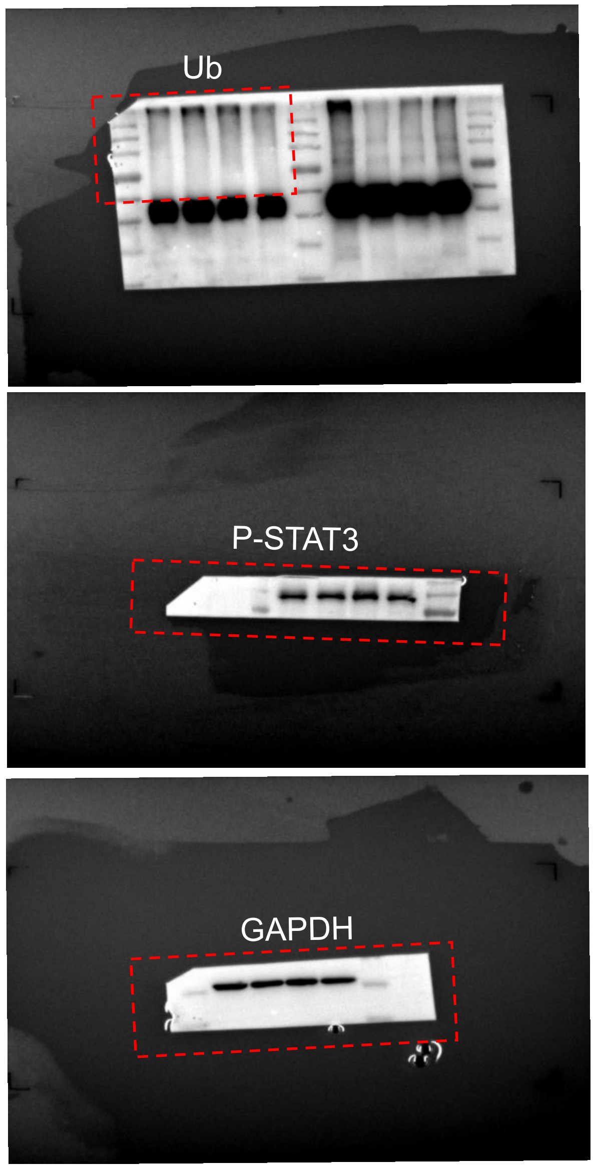

Supplement: Supplementary file 1 — Supplemental Material [file 41419_2025_7358_MOESM1_ESM.pdf]
